# Supplementary figures and images for: Label-free imaging and classification of live P. falciparum enables high performance parasitemia quantification without fixation or staining
Source: PLoS Comput Biol. 2021 Aug 9;17(8):e1009257. doi: 10.1371/journal.pcbi.1009257 (PMC8376094; doi:10.1371/journal.pcbi.1009257)

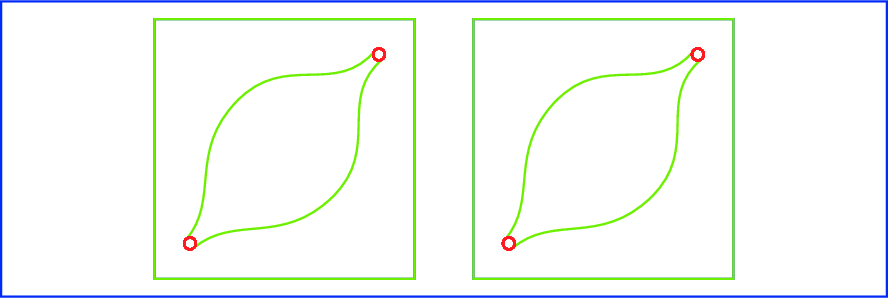

Supplement: S1 Fig — 1 mm circular holes (shown in red below) were laser-cut into quartz slides (Ted Pella 26011, Redding, USA) outlined in blue, using a ULS-P150D laser-cutting system (Universal Laser Systems, Scottsdale, USA). Gaskets (green) were laser-cut from Parafilm (Sigma Aldrich P7793, St. Louis, USA). After cutting, gaskets were aligned by hand, then pressed down gently around the exterior. Quartz coverslips (Ted Pella 26014) were aligned by hand to the gaskets, and then pressed gently. The assembly was sealed by compression of the flow cell (protected inside a single layer aluminum foil envelope) with a mass of 300 g for five minutes, on a hot plate set to 75°C. (TIF) [file pcbi.1009257.s001.tif]

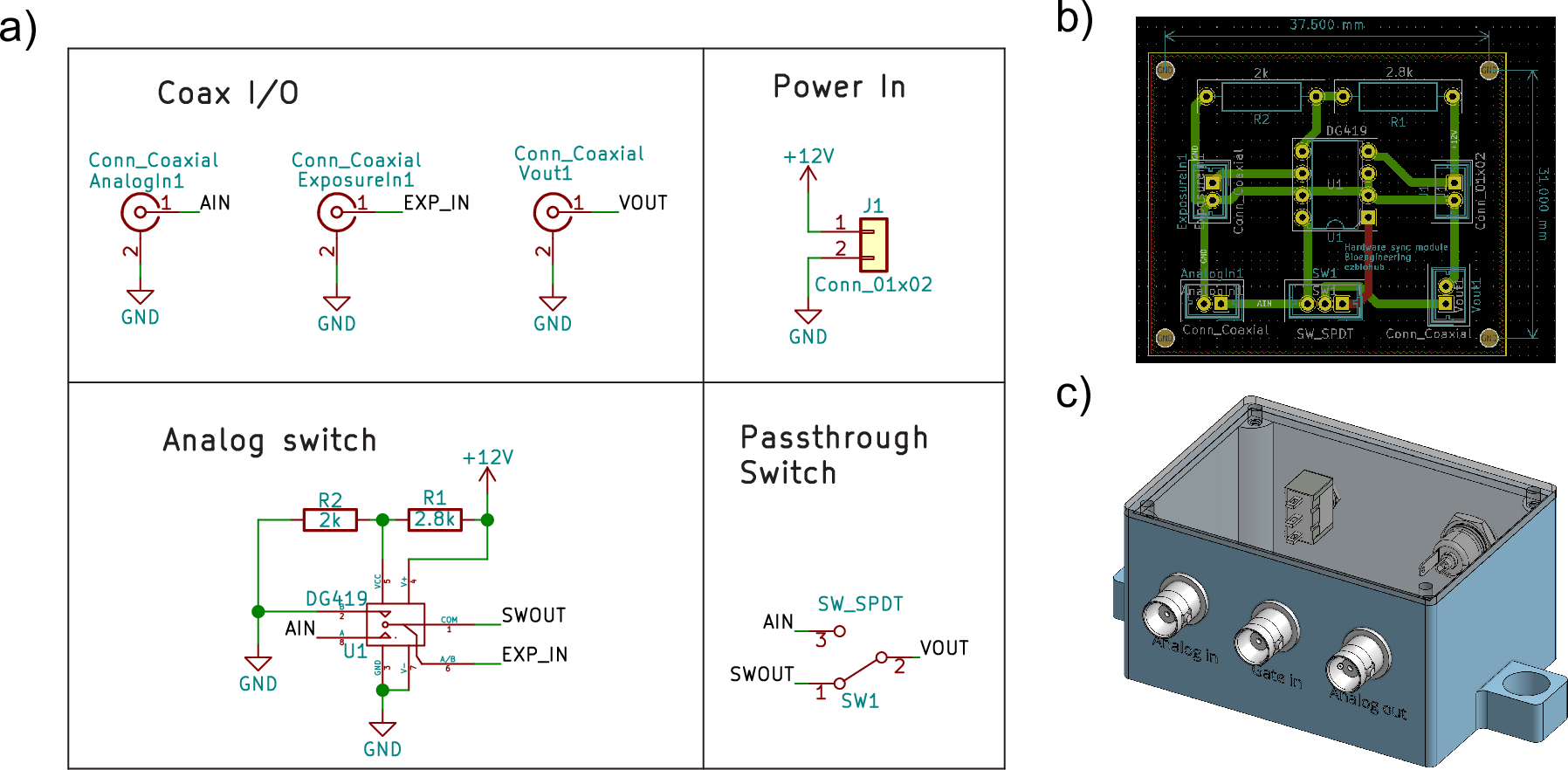

Supplement: S2 Fig — a) Schematic of the simple circuit used to synchronize LED emission with camera exposure. The circuit uses an analog switch to connect either a programmable analog voltage signal (AIN) or ground (GND) to the output, gated by the camera exposure’s logic signal. In series with the analog switch is a manual toggle switch to optional bypass the analog switch, disabling the sync module. b) PCB layout diagram for the hardware sync module. Note that coaxial connector footprints were replaced with standard 0.1” headers, and connected via wires to panel-mount BNC connectors. The circuit was fabricated as a PCB using an LPKF Protomat S103 circuit mill (LPKF, Garbsen, Germany). c) Solid model screenshot of the 3D-printed enclosure for the hardware sync module. (TIF) [file pcbi.1009257.s002.tif]

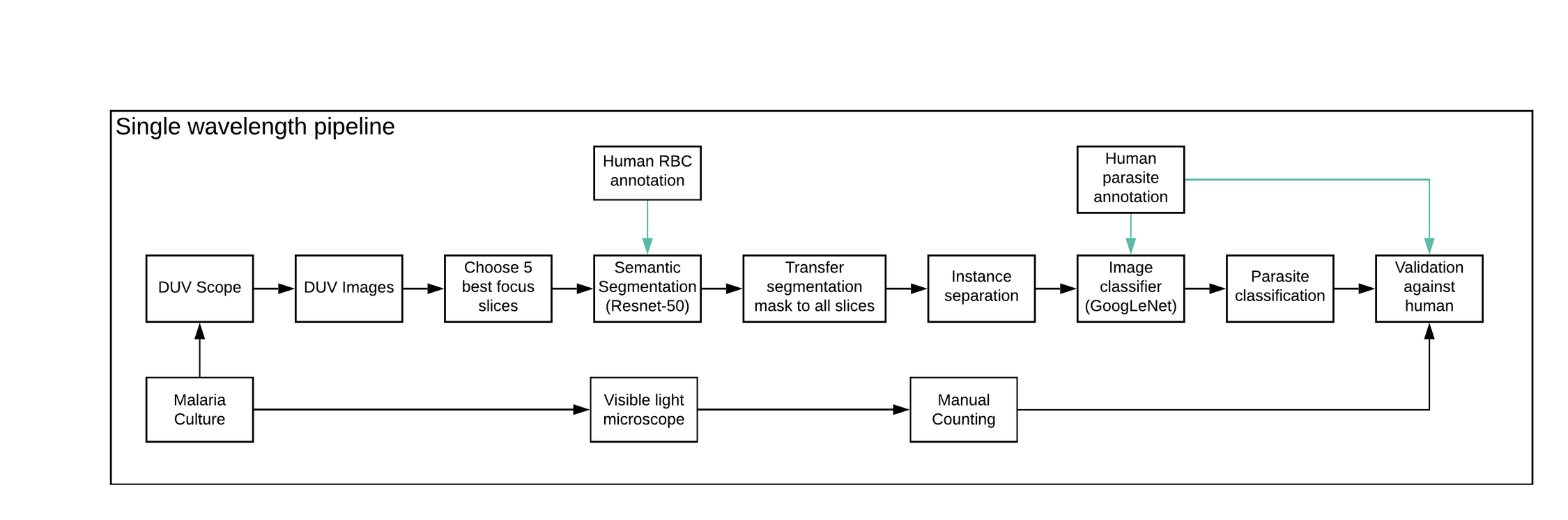

Supplement: S3 Fig — (TIF) [file pcbi.1009257.s003.tif]

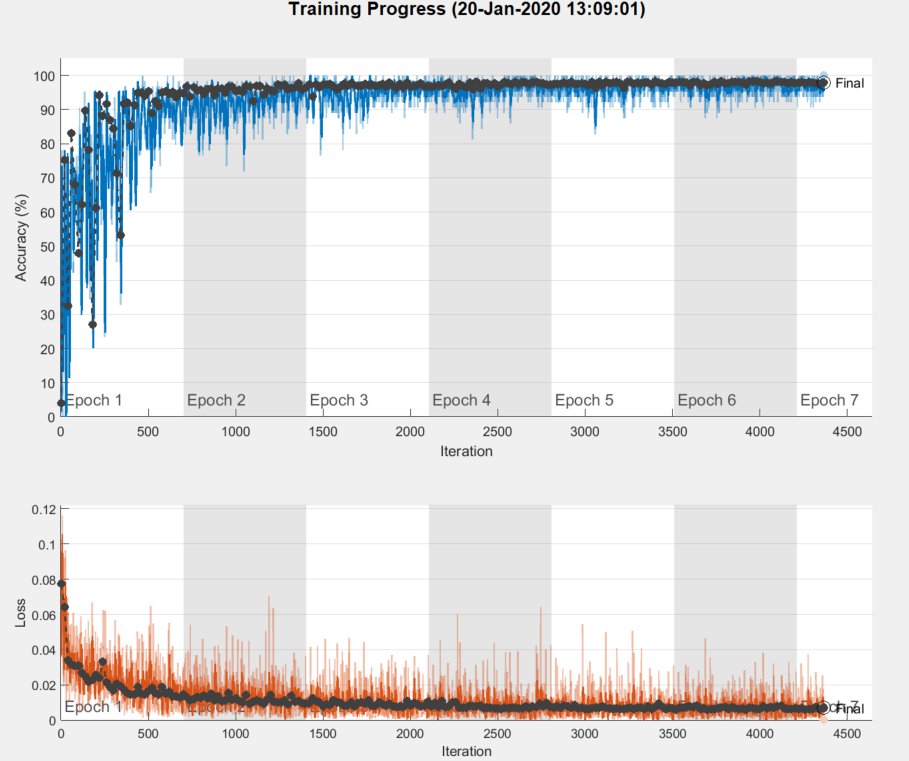

Supplement: S4 Fig — The upper plot shows the overall accuracy as a function of iteration and epoch number. The blue line plot (no markers) shows training dataset accuracy while the black line plot (dashed with circle markers) shows validation accuracy. The lower plot shows the loss function (red with no markers: training loss; black with circle markers: validation loss. (TIF) [file pcbi.1009257.s004.tif]

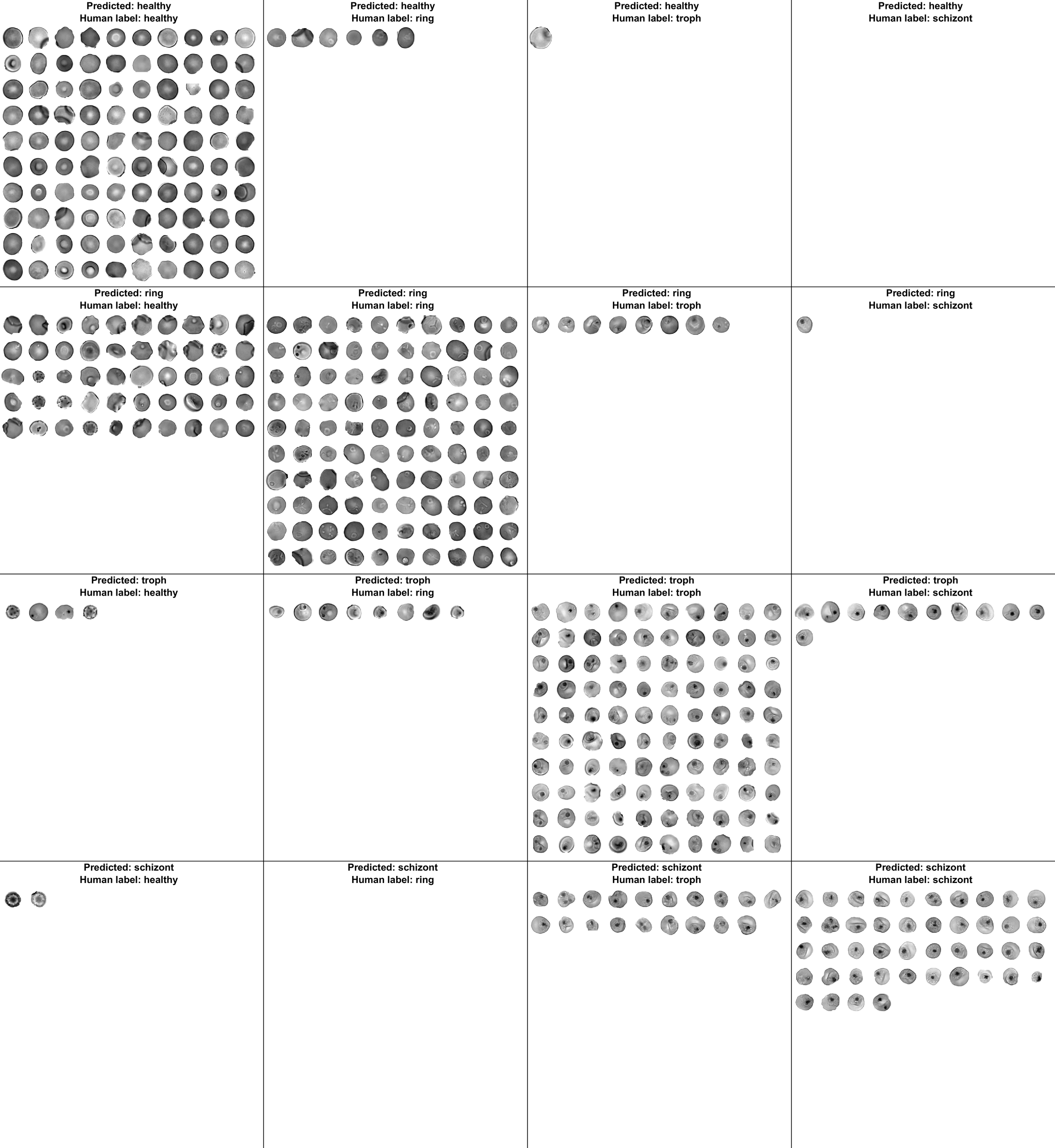

Supplement: S5 Fig — The arrangement of the categories is the same as Fig 2 from the main text. For this montage, a maximum array size of 10 x 10 was used for display. Note that for many of the categories (especially off-diagonal entries) there were not enough examples to populate the array, in which case the space was left blank. All data from this figure was taken from the validation dataset, as opposed to the training dataset. (TIF) [file pcbi.1009257.s005.tif]

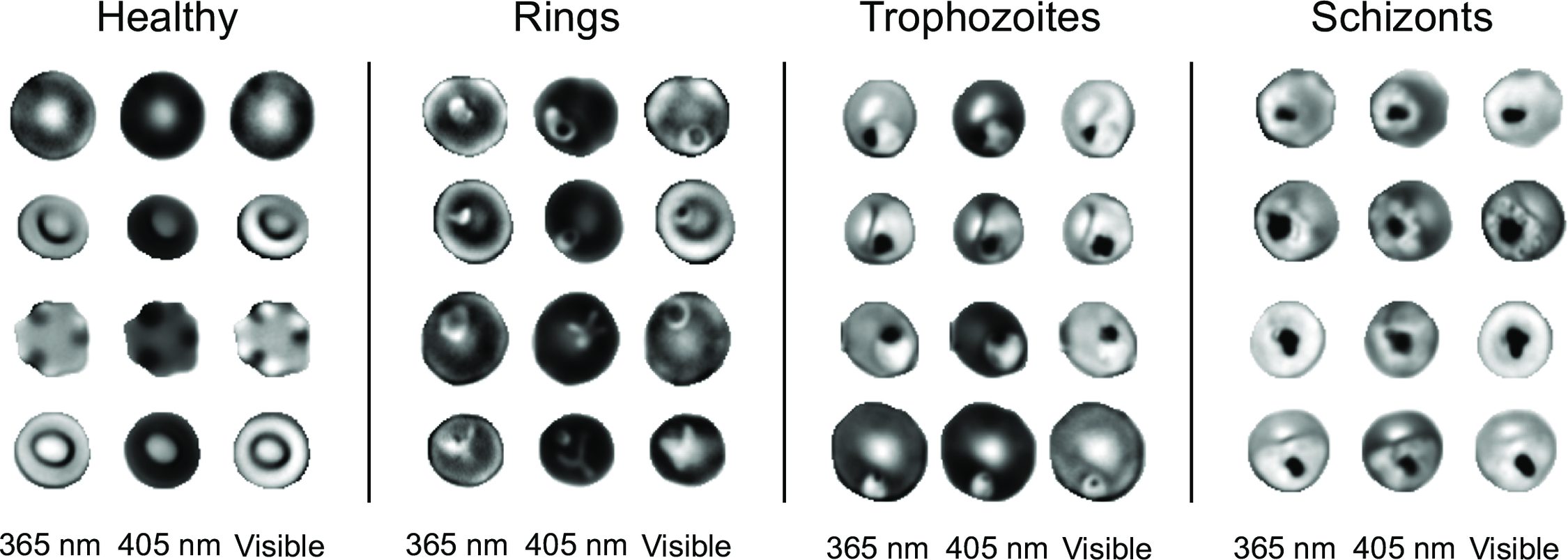

Supplement: S6 Fig — (left columns: 365 nm, middle columns: 405 nm, right columns: standard visible LED). Four example cells are displayed for each category, where each row within a category are images of the same physical cell. Ring stage parasites are seen to be highly mobile, as evidenced by their differing positions and even morphology between acquisitions at different wavelengths, which were separated in time on the order of a few minutes. (TIF) [file pcbi.1009257.s006.tif]

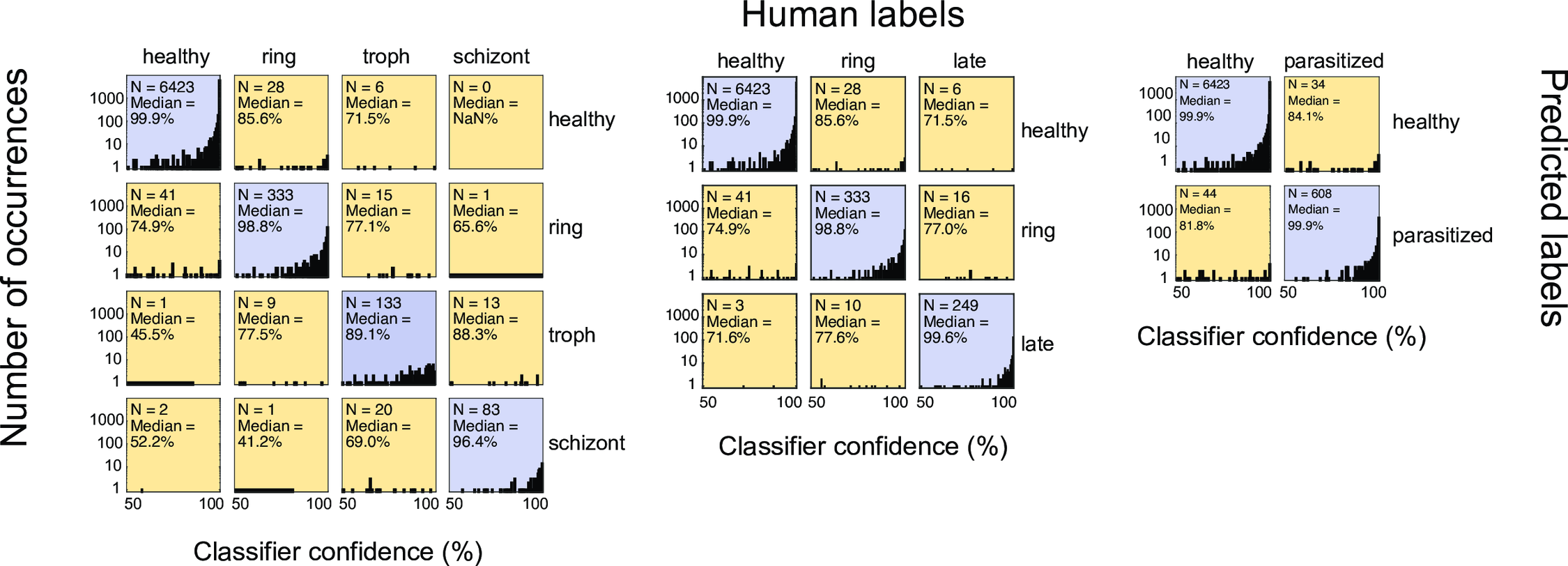

Supplement: S7 Fig — In all three plots, log-scaled histograms of classifier confidence scores are displayed in the pattern of a confusion matrix. Statistics are derived by evaluating the validation dataset from the 285 nm classifier. Total instance counts for each matrix element as well as the medians of the distributions are shown as text insets. (TIF) [file pcbi.1009257.s007.tif]

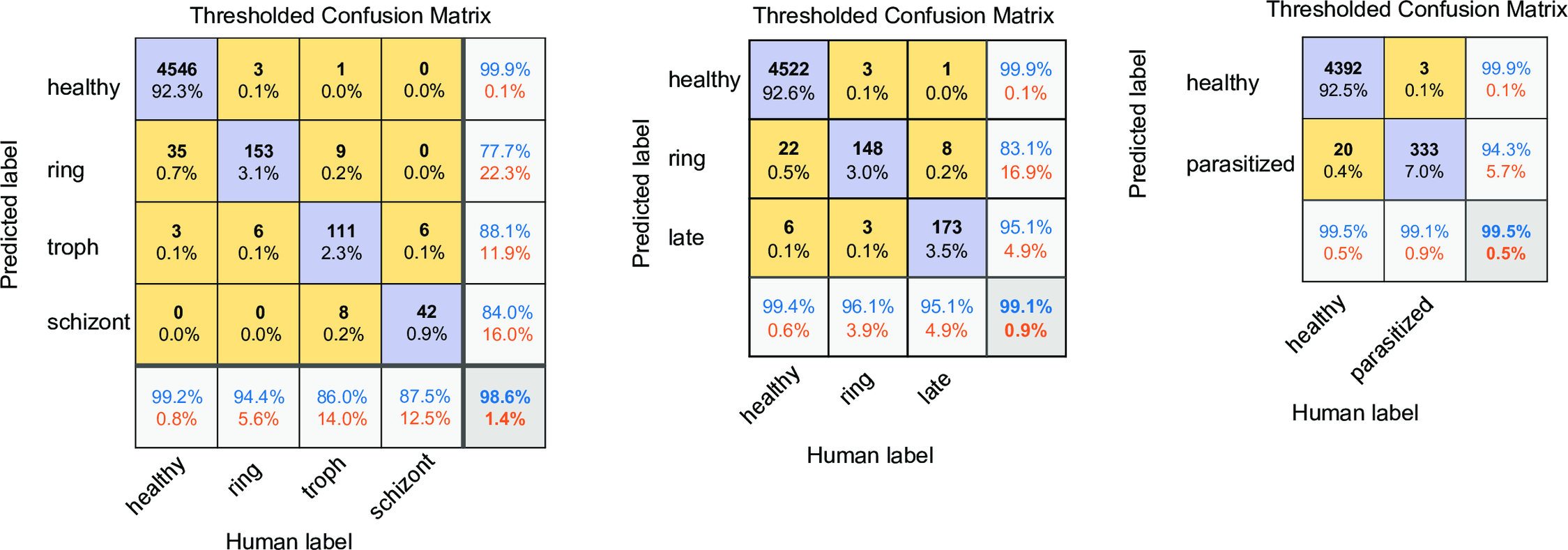

Supplement: S8 Fig — These confusion matrices show the result of applying the empirically-optimal threshold values of 61%, 76%, and 96%, to the four, three, and two-category classifier confusion matrices. (TIF) [file pcbi.1009257.s008.tif]

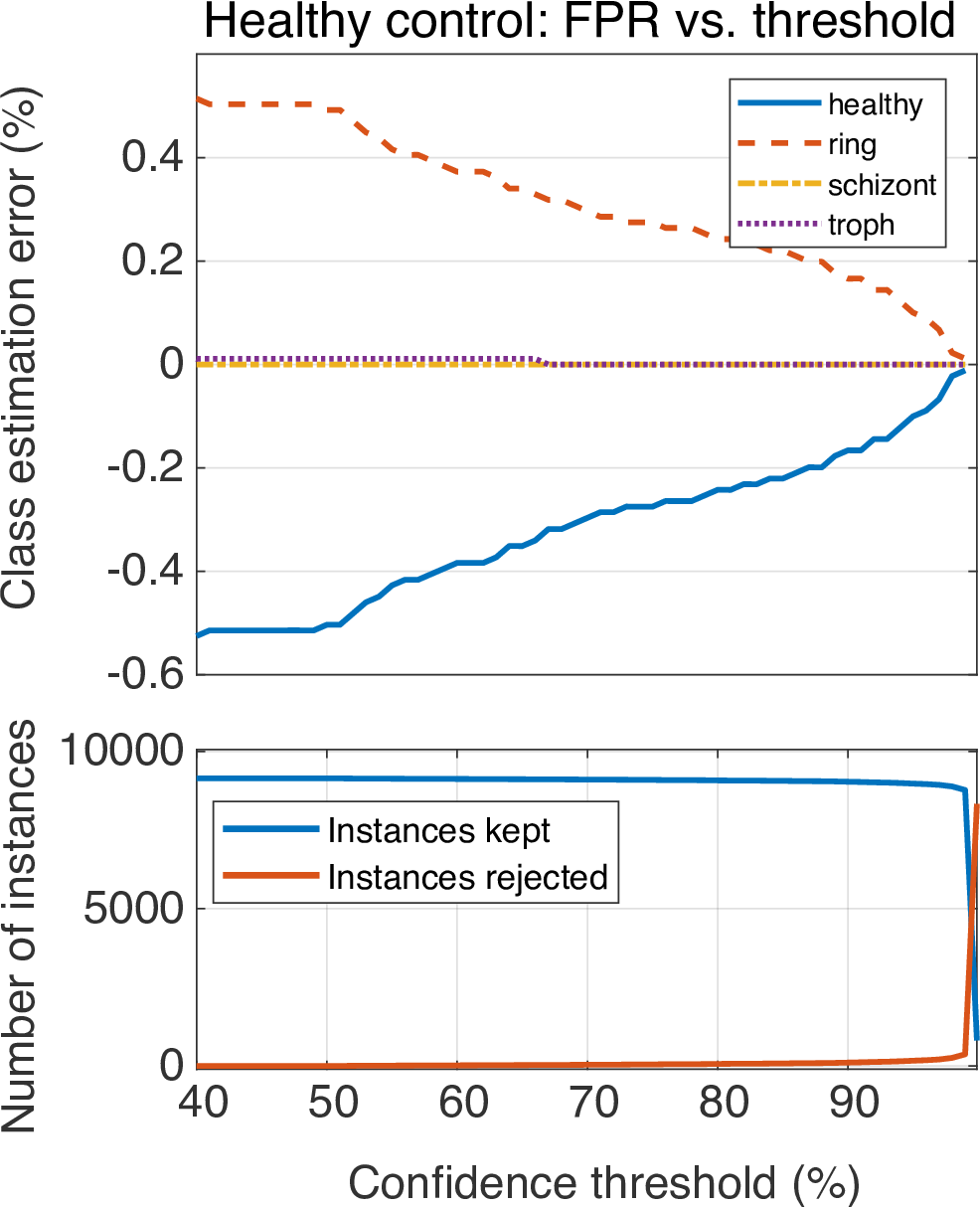

Supplement: S9 Fig — Top: Sample composition estimates are shown as a function of confidence threshold, demonstrating that the approximately 0.5% raw FPR can be reduced by rejecting cells with low classifier confidence. Bottom: The number of cells kept (blue) and rejected (red) by the thresholding process are plotted as a function of threshold value. (TIF) [file pcbi.1009257.s009.tif]

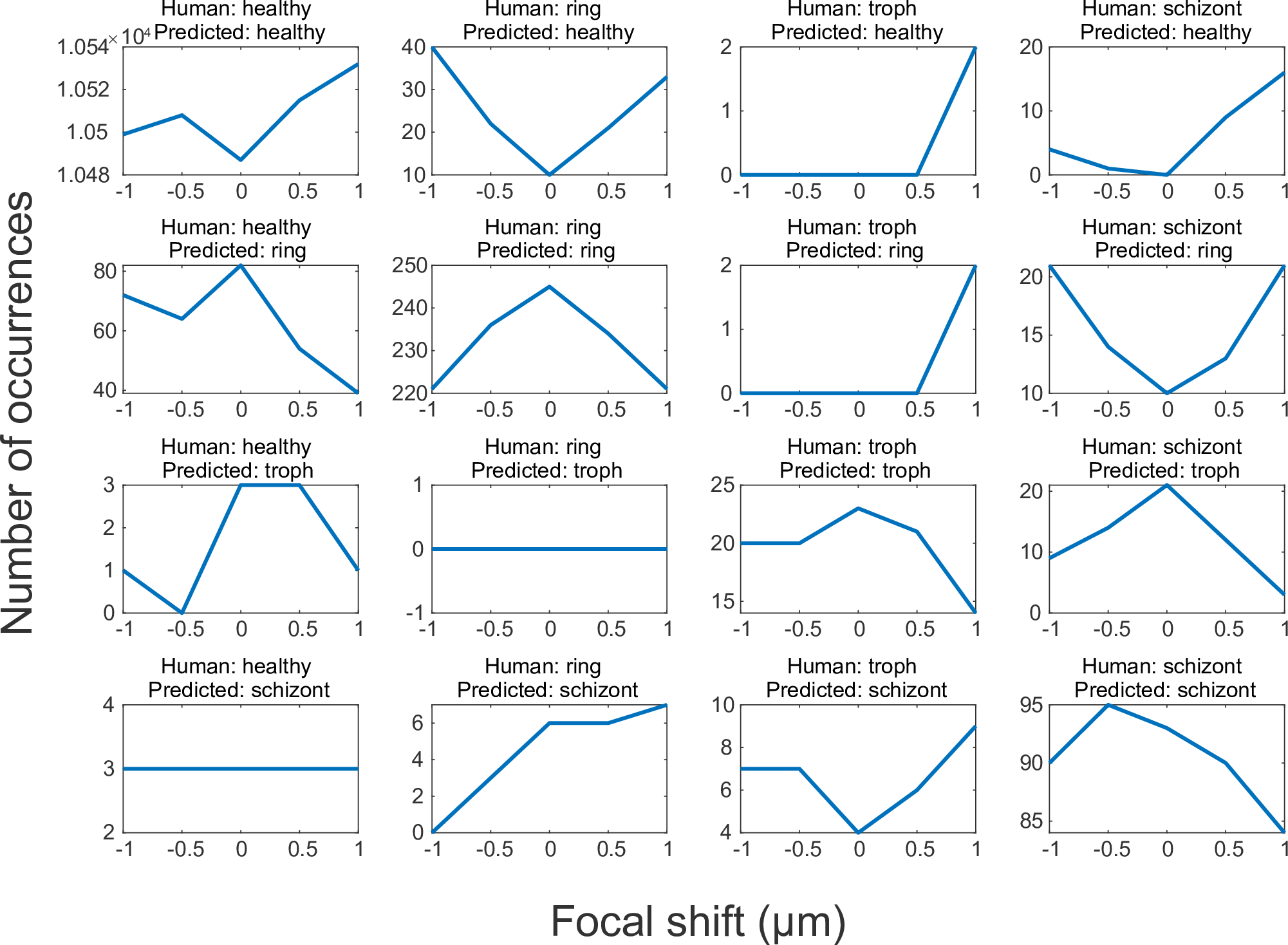

Supplement: S10 Fig — All panels spatially correspond to confusion matrix elements from Fig 2, and display relative changes in matrix values as a function of defocus. Diagonal matrix entries (true positives) corresponding to all three parasite life cycles stages tend to improve with global best focus, while many off-diagonal entries (false negatives / false positives) see reductions at the global best focus. However, exceptions include ring false positives (second row, first column) and trophozoite/schizont cross-identification. (TIF) [file pcbi.1009257.s010.tif]

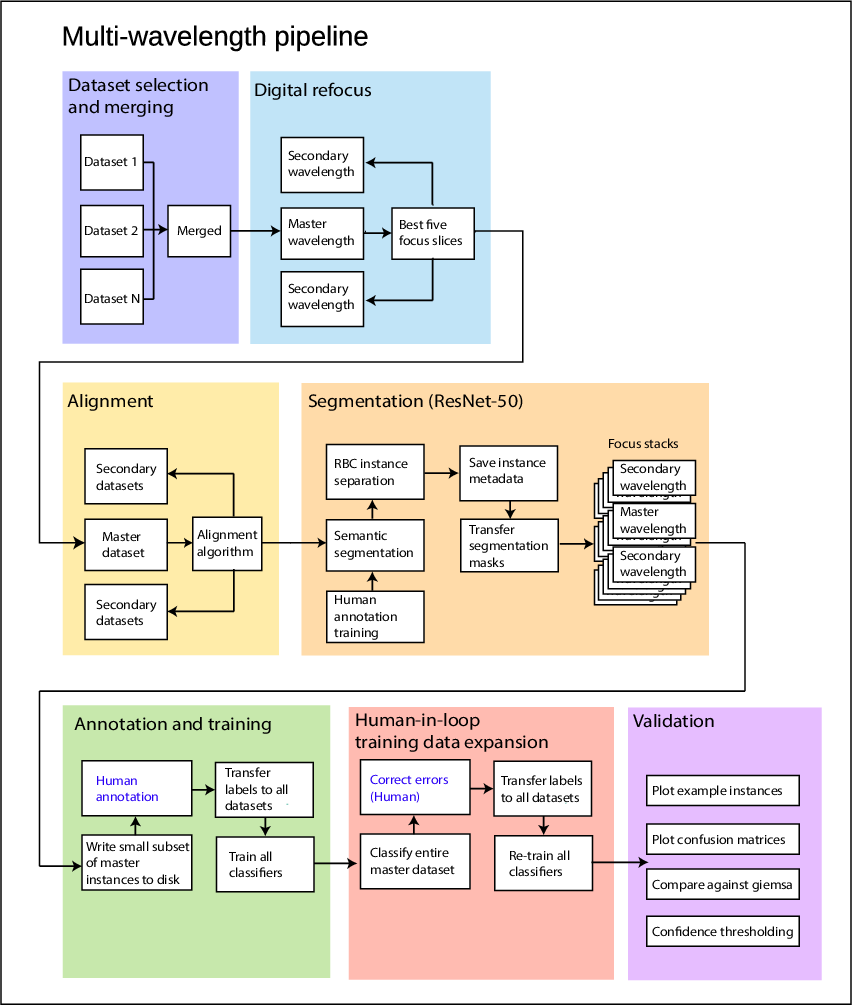

Supplement: S11 Fig — 1. Load data structures (UV scope) or raw images (commercial scope). 2. Apply slice selection. 3. Register wavelength channels using affine transformation. 4. Export pre-processed images. 5. Do semantic segmentation on the master wavelength/slice using ResNet-50. 6. Apply instancing algorithm to all wavelengths/slices, exporting RBC instances to disk. 7. Classify all RBC instances for only the master wavelength and slice. 8. Apply the resulting labels to all the corresponding instances in the other slices/channels. 9. Export the lowest-confidence subset of the machine-classified images for human annotation. 10. Import the results of human annotation and propagate labels to all the other channels/slices. 11. Train new classifiers with the high confidence dataset. (TIF) [file pcbi.1009257.s011.tif]

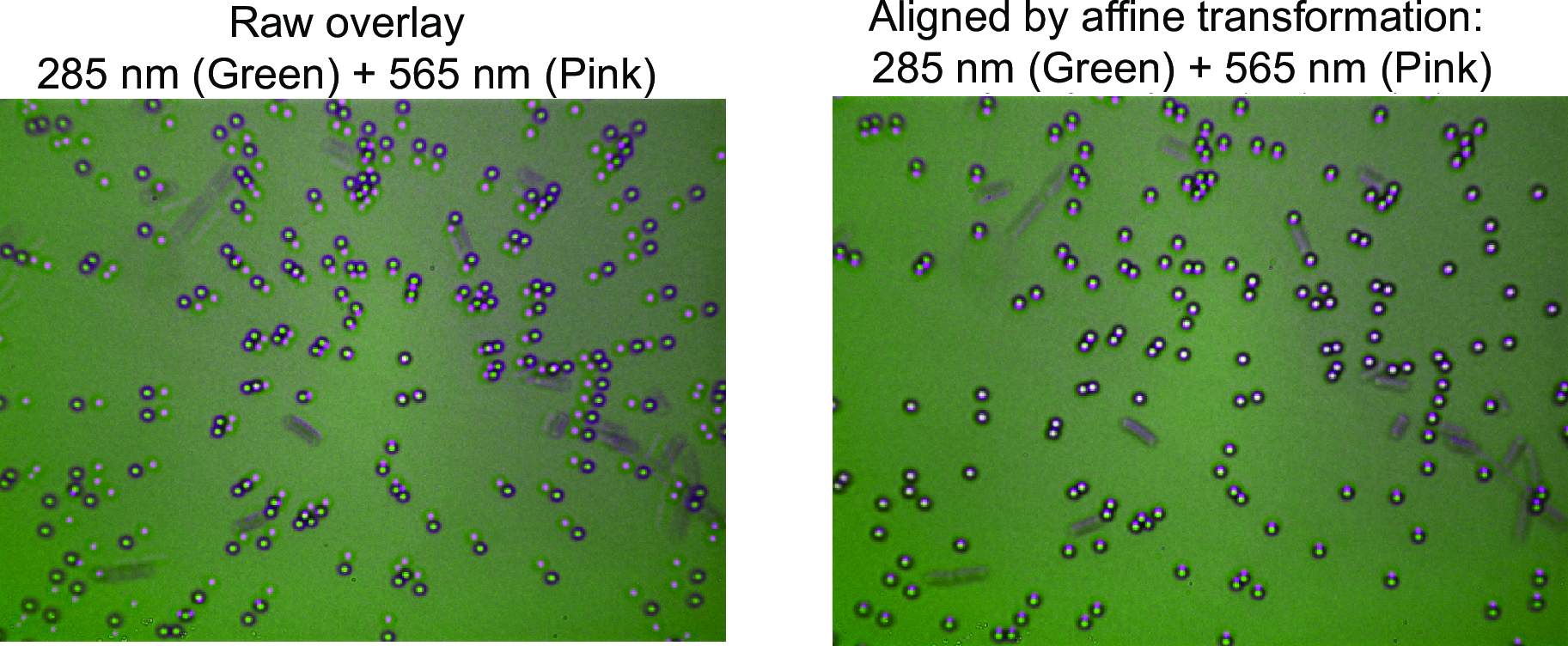

Supplement: S12 Fig — Left: overlay of two raw images from the UV scope, acquired at 285 nm (green) and 565 nm (pink). Right: overlay of the same images after image registration was completed. The sample in the images consists of fixed E. Coli cells (rods) mixed with 1 μm beads (circles). (TIF) [file pcbi.1009257.s012.tif]

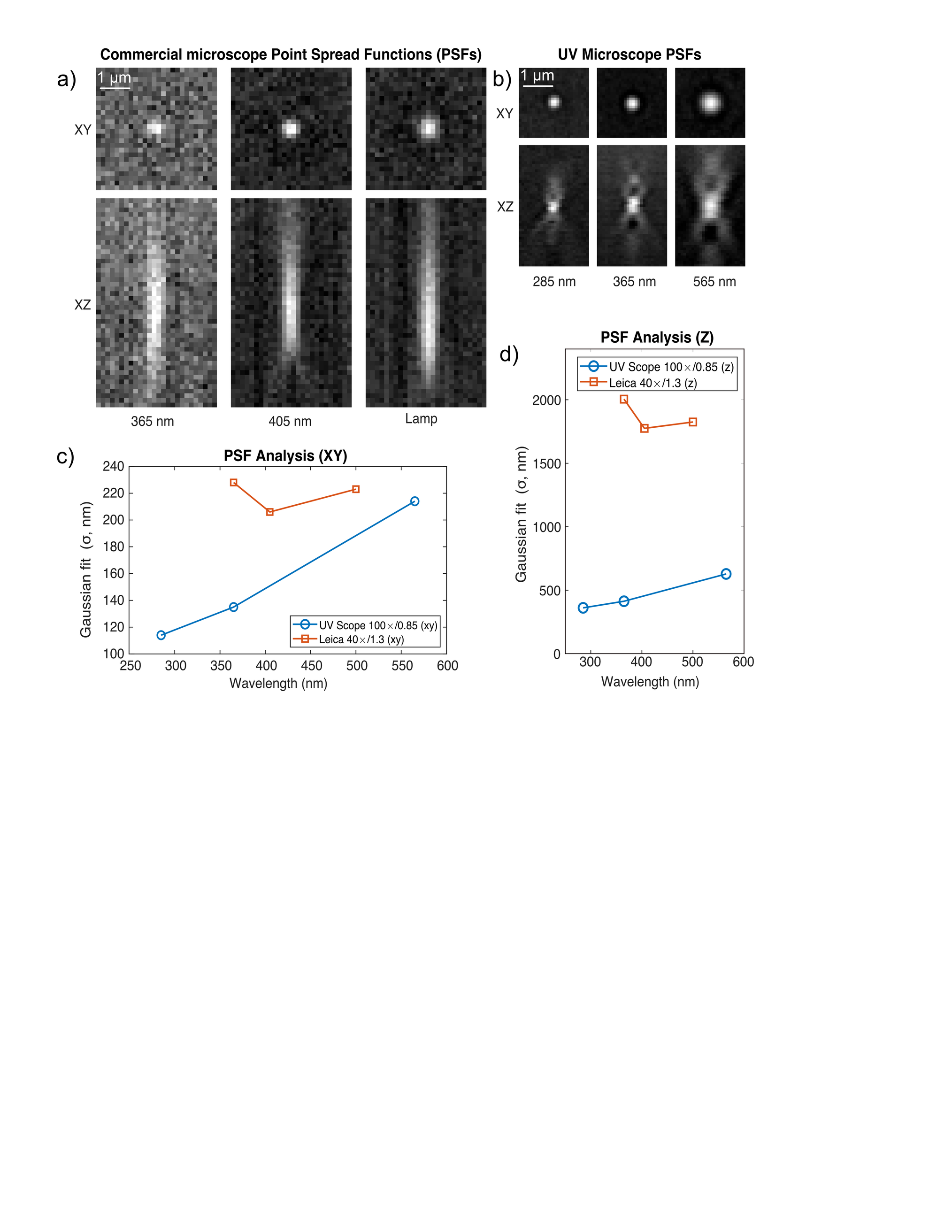

Supplement: S13 Fig — PSFs were acquired in the brightfield modality by imaging 100 nm gold nanoparticles adhered to the surface of a 0.25 mm thick quartz coverslip. All images in a) and b) are shown at the same spatial scale such that sample plane distance is directly comparable between images. a) Commercial 40 × /1.3 microscope PSFs at 365, 405, and white LED. PSFs display substantial spherical aberration as evidenced by elongated axial depth of focus and broader in-plane width, likely resulting from a mismatch in coverslip thickness. b) UV Scope PSFs acquired at 285, 365, and 565 nm, exhibiting diffraction-limited widths, both in-plane and axially. c) Summary of lateral PSF widths, as approximated by Gaussian fits. d) Summary of axial widths, as approximated by Gaussian fits. (TIF) [file pcbi.1009257.s013.tif]

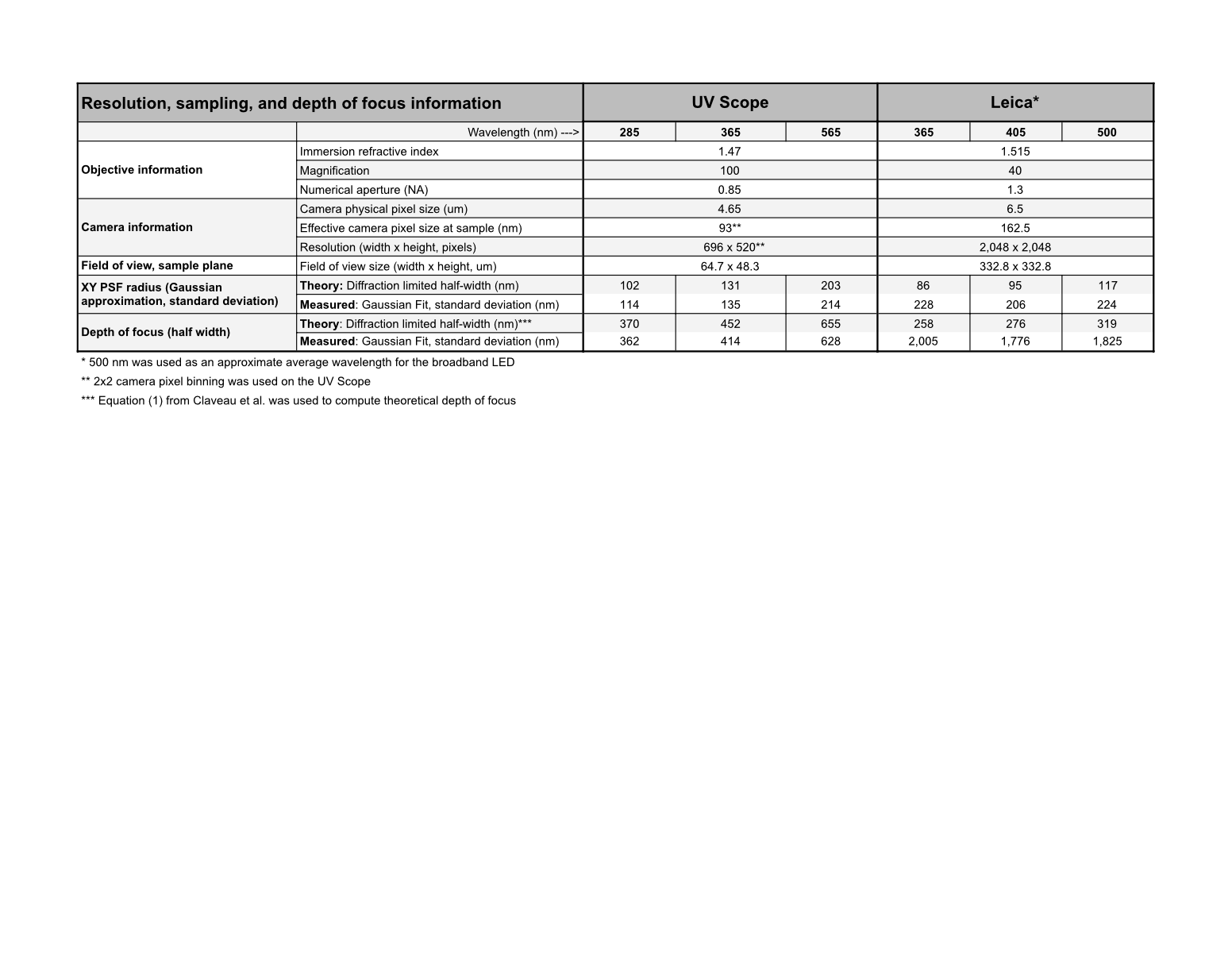

Supplement: S14 Fig — (TIF) [file pcbi.1009257.s014.tif]

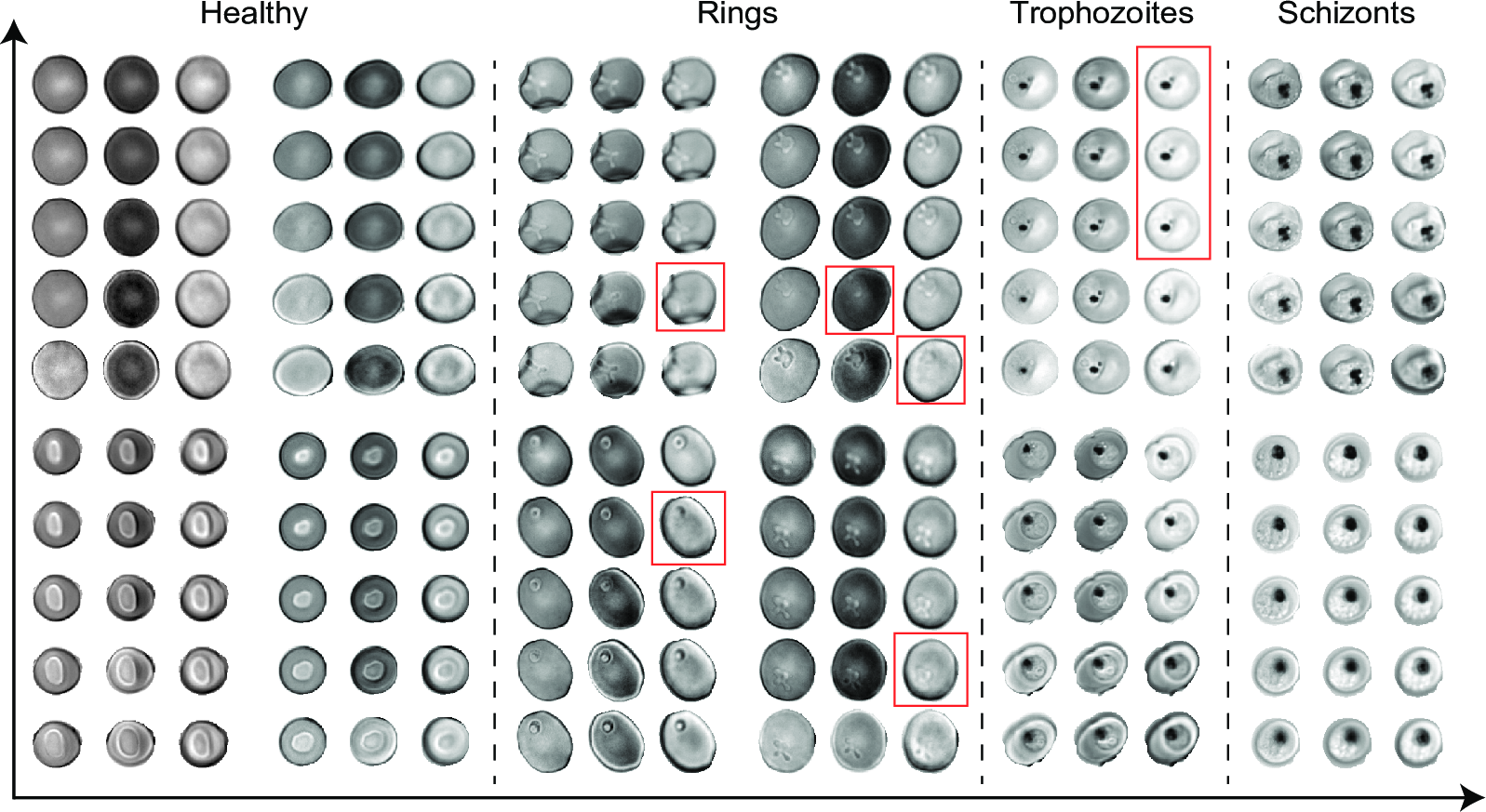

Supplement: S15 Fig — For all panels, the color/focus layout is identical to Fig 4. Examples of all four categorized life cycle stages are shown. (TIF) [file pcbi.1009257.s015.tif]

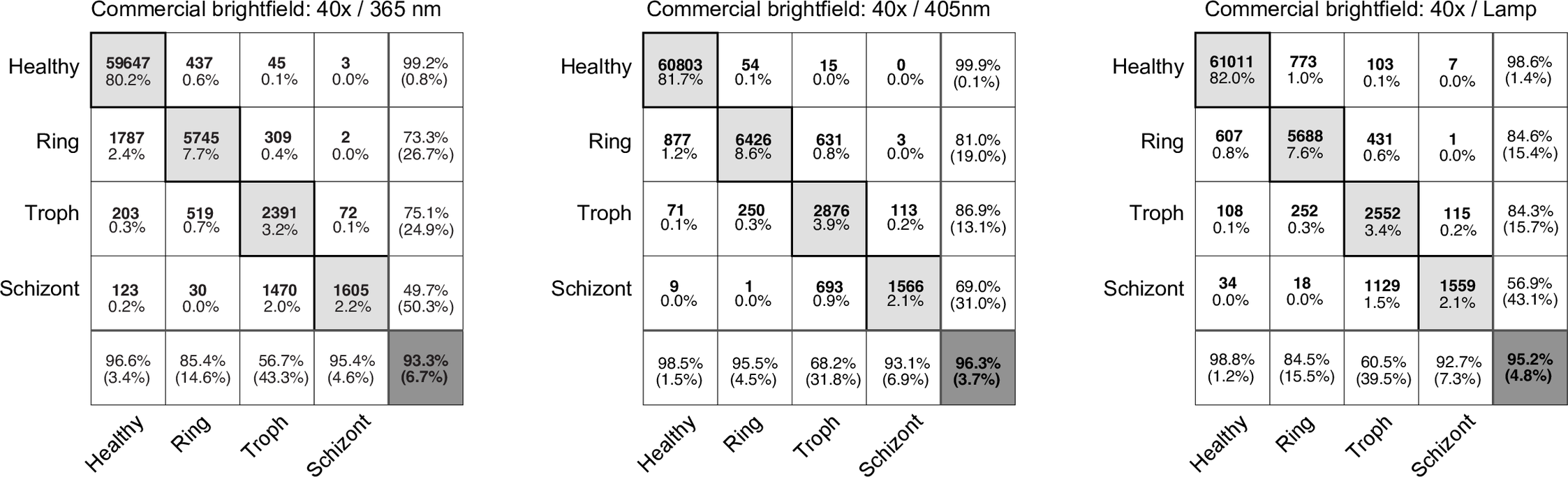

Supplement: S16 Fig — Left: 365 nm, Middle: 405 nm, Right: Visible light LED. All classifiers were trained on the identical set of RBC instances, using images acquired each at their respective wavelength (multiwavelength dataset, see Table 1). (TIF) [file pcbi.1009257.s016.tif]

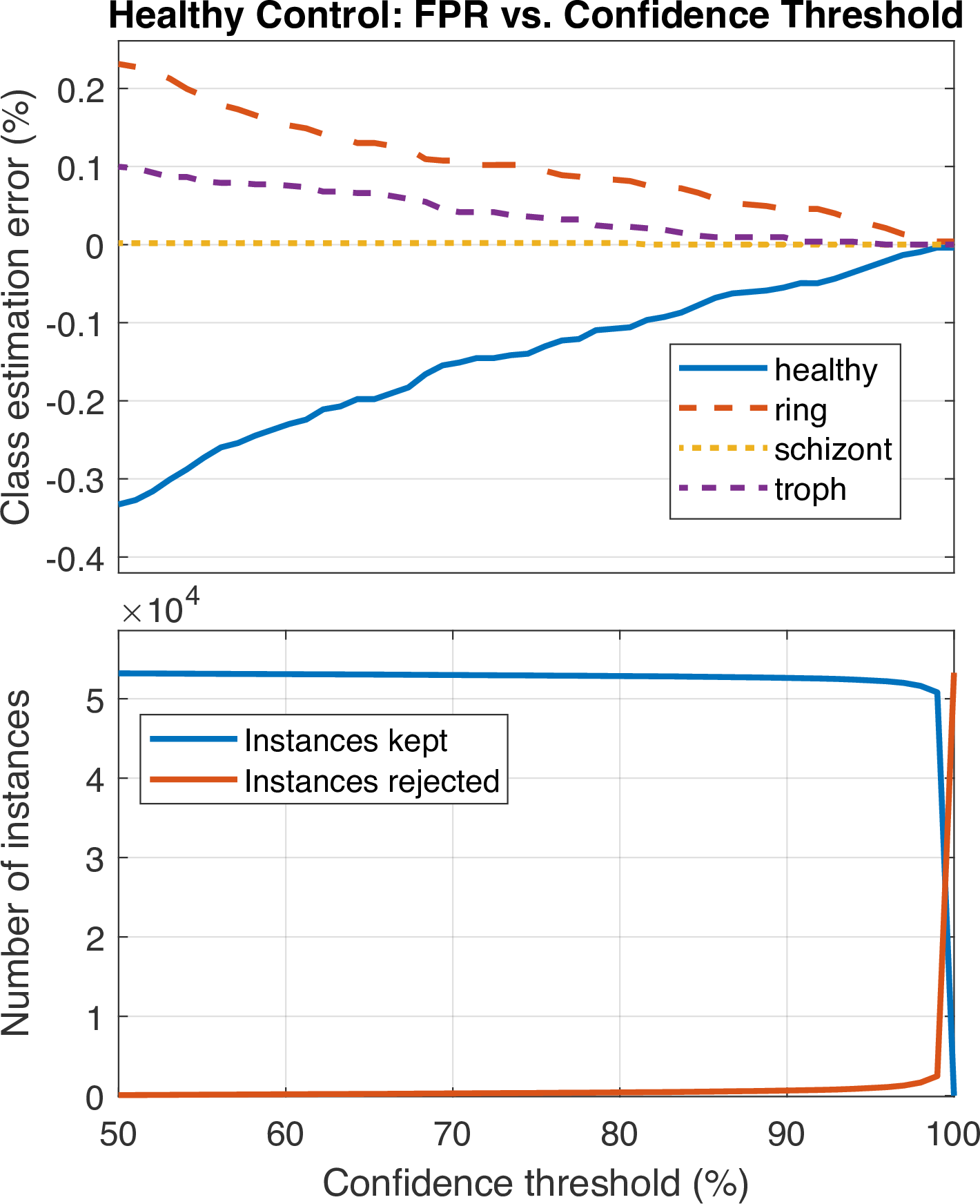

Supplement: S17 Fig — The upper plot displays the sample composition error for all four stages as a function of confidence threshold. Data processed were from a healthy donor sample, uninfected by Plasmodium. In particular, the FPR for rings at 50% confidence threshold is substantially lower than the results reported in Fig 5. We explain this by noting this particular healthy control sample exhibited very few echinocytes, to which we attribute the low basal FPRs as compared to Fig 5. (TIF) [file pcbi.1009257.s017.tif]

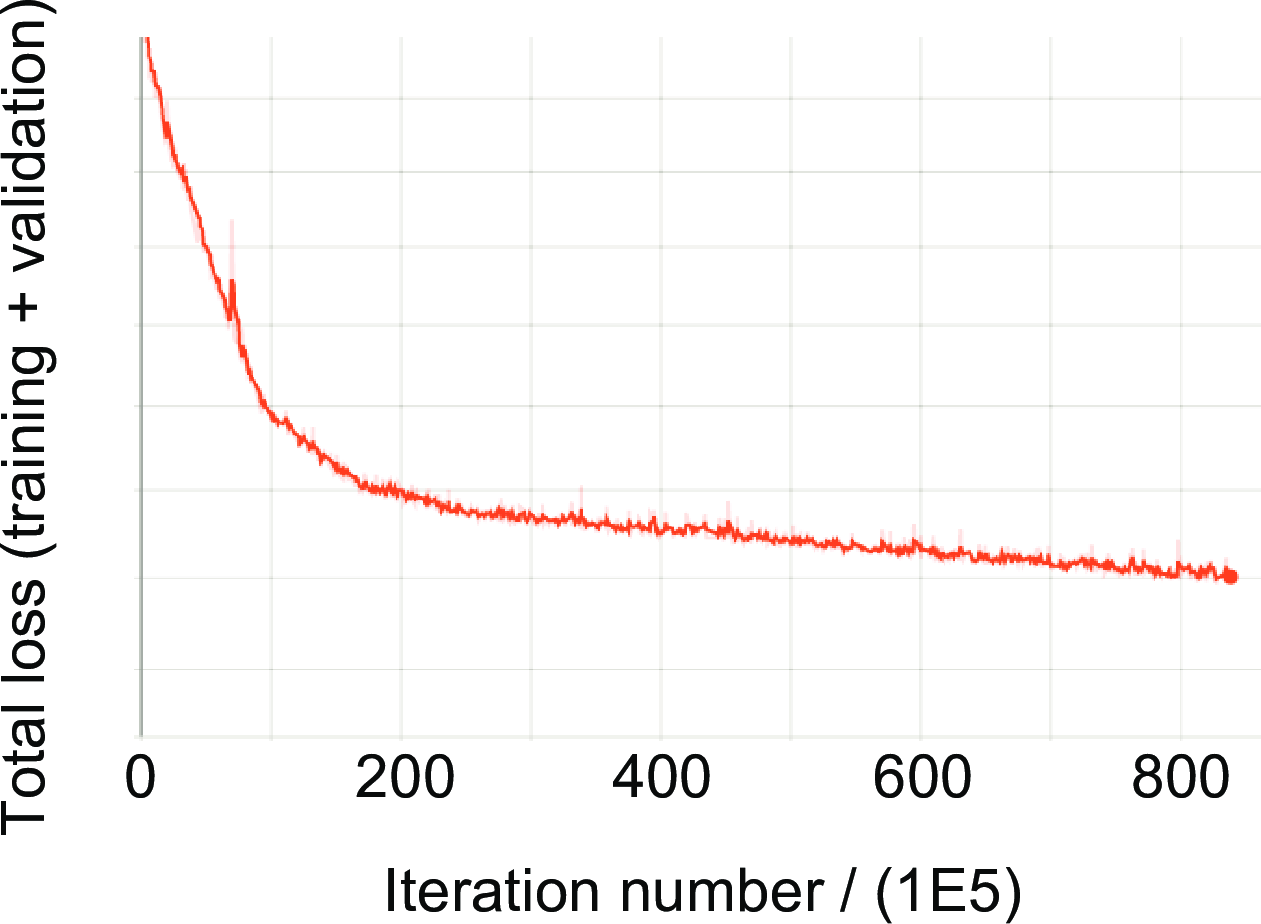

Supplement: S18 Fig — (TIF) [file pcbi.1009257.s018.tif]

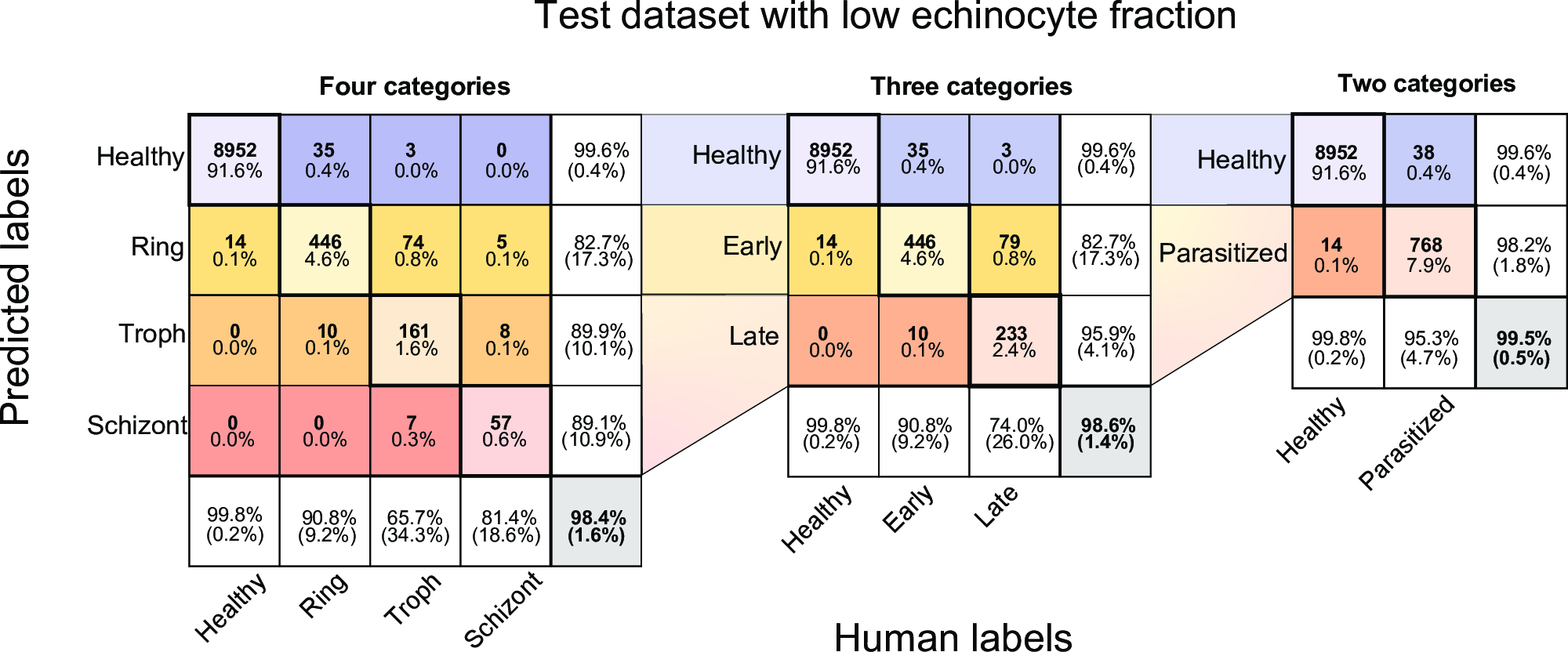

Supplement: S19 Fig — As compared with Fig 2, the FPR for rings is reduced presumably due to fewer cells containing anomalous morphology, and is in closer agreement to the result extracted by least squares fit to the titration data in Fig 8. The random sub-partition was configured to contain overall parasite prevalence similar to Fig 2. (TIF) [file pcbi.1009257.s019.tif]

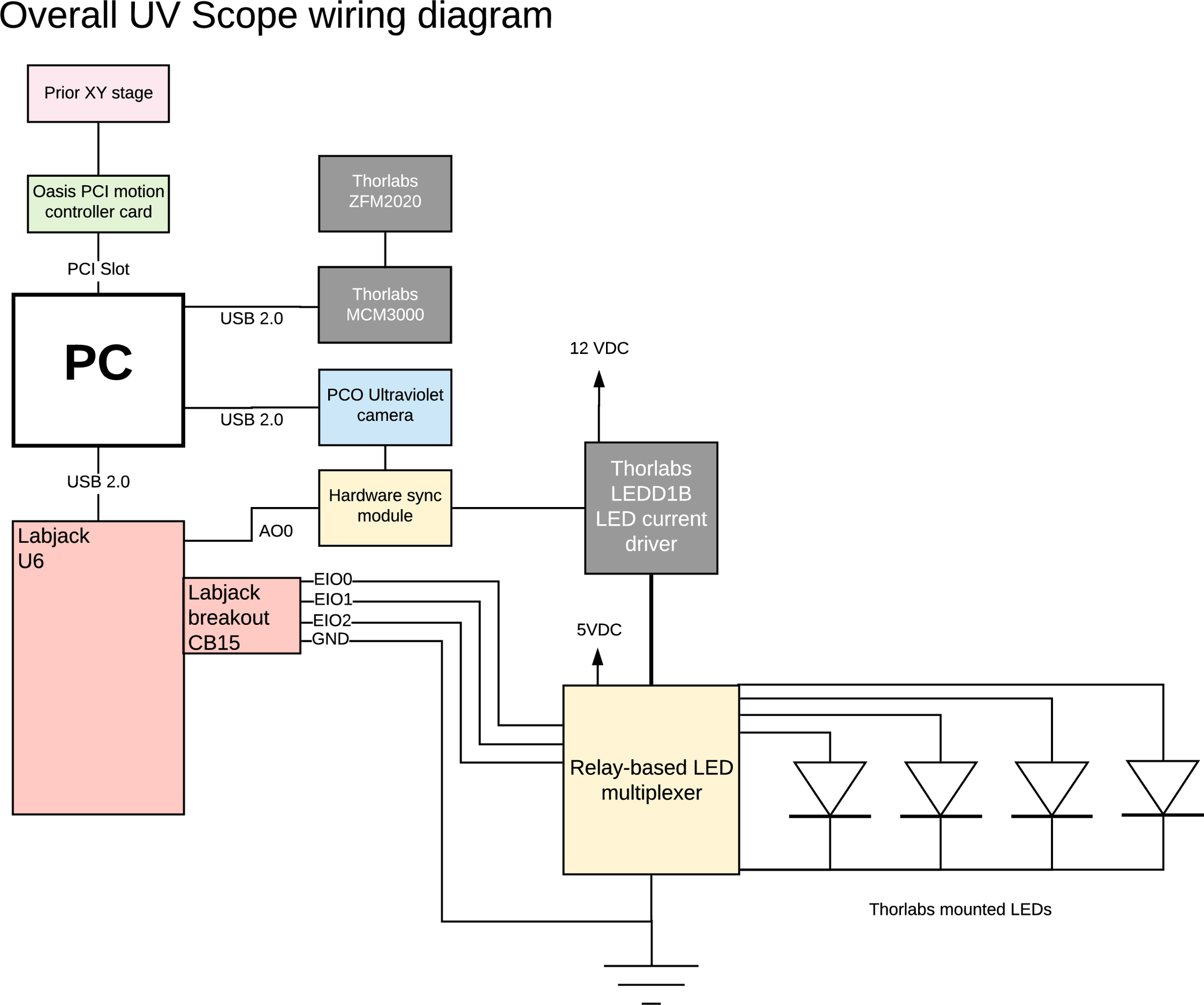

Supplement: S20 Fig — The microscope hardware was controlled by a centralized PC running Windows 10. All the various hardware devices were controlled by a combination of standard and custom Matlab classes, as described in S22 Fig. Other specific hardware configurations are possible with minor changes to the software. (TIF) [file pcbi.1009257.s020.tif]

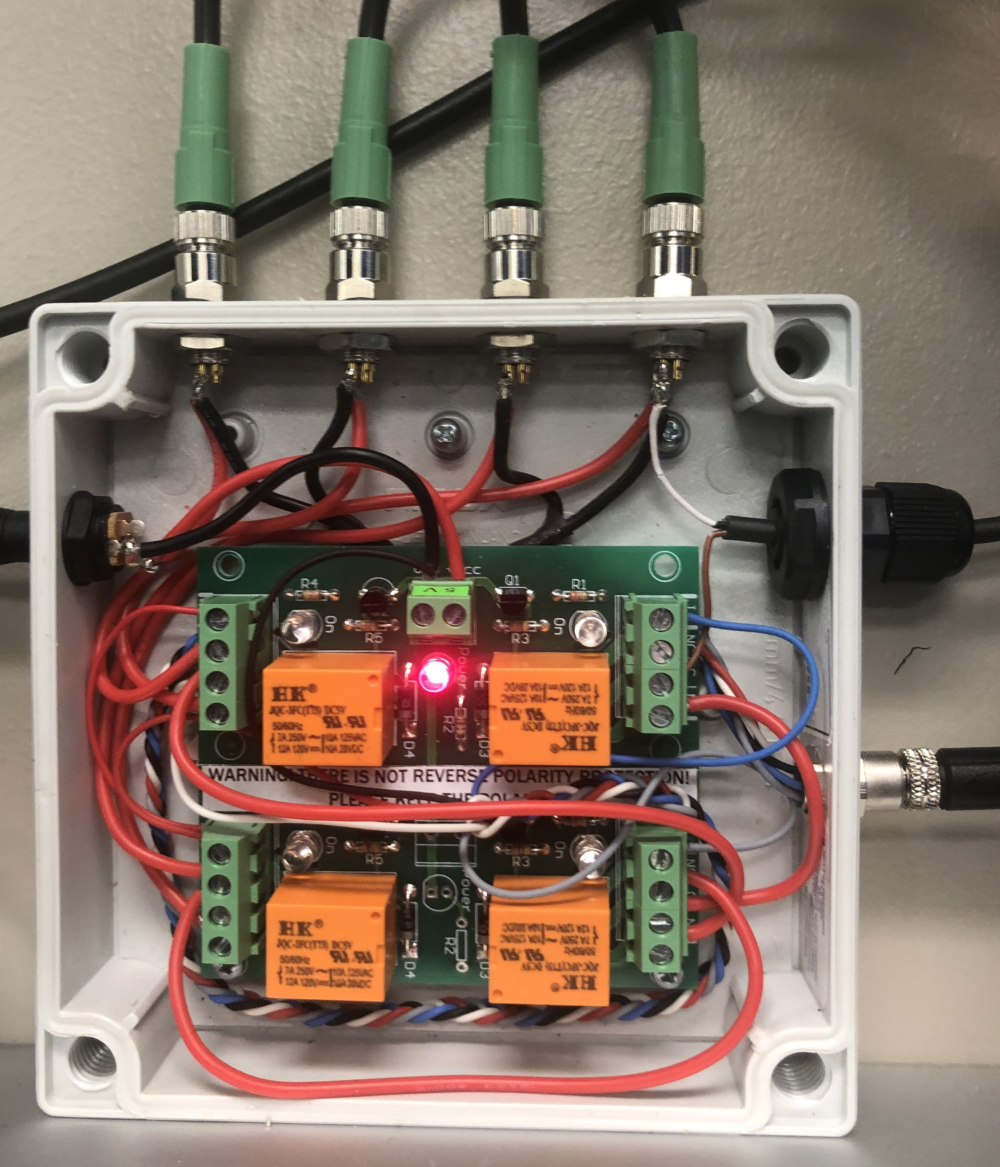

Supplement: S21 Fig — The LED multiplexer uses a four-channel relay (Denkovi DAE-RB/Ro4-JQC-5V, Byala, Bulgaria) to route the current generated by a Thorlabs LED current driver (LEDD1B) to one of four LEDs. The relay board was mounted inside a box to house the wired connections between relays. The box received a three-pin signal (plus GND). The first relay was used as an overall ON/OFF switch, while the remaining three were arranged in a binary tree to route current from the driver to any of the four LEDs. (TIF) [file pcbi.1009257.s021.tif]

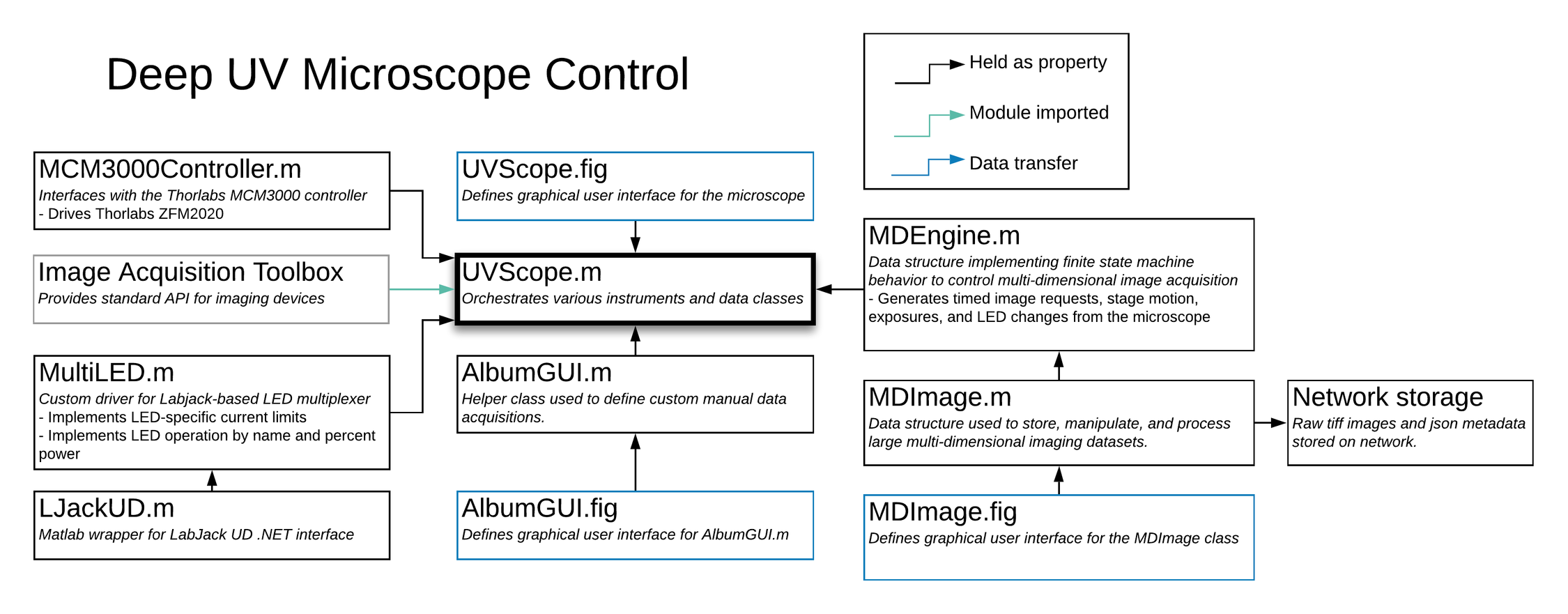

Supplement: S22 Fig — A set of custom Matlab classes were used to configure and execute multi-dimensional image acquisitions. Multiplexed LED illumination was driven by a data class which maps maximum allowable LED currents to control voltages, sending a digital address to the relay multiplexer for LED selection, and an analog voltage representing percent power. The MDEngine class implements the conversion from acquisition parameters to active control of the UVScope, including real-time focus tracking across large sample areas, in order to keep focal stacks centered on the sample. All microscope control software can be found at: https://github.com/czbiohub/UVScope-control. (TIF) [file pcbi.1009257.s022.tif]
