# Supplementary material for: Label-free imaging and classification of live P. falciparum enables high performance parasitemia quantification without fixation or staining
Source: PLoS Comput Biol. 2021 Aug 9;17(8):e1009257. doi: 10.1371/journal.pcbi.1009257 (PMC8376094; doi:10.1371/journal.pcbi.1009257)
Supplement: Attachment — Submitted filename: Editor and reviewer responses.pdf [file pcbi.1009257.s024.pdf]

Label-free imaging and classification of live *P. falciparum*

Dear Editor,

Thank you very much for the invitation to re-submit this manuscript, and we also thank the reviewers for the time they have taken from their schedules to help us improve the quality of our work. Please find our itemized responses to all specific points detailed below in blue, with editor and reviewer comments shown in black:

Editorial feedback:

In relation to comments by reviewer one in relation to the AI, I would encourage to make these points in the discussion of the novelty vs. the implementation novelty of the approach and how it relates to the current state-of-the-art in AI for medical image analysis.

We agree that it is important to be as clear as possible about exactly where the innovation in this work lies with respect to existing work in the field. We have made wording changes to both the introduction and discussion, highlighting our contribution as a novel implementation of existing technology. It is also likely that current and future advances in machine learning may further improve the performance of the method.

In relation to reviewer 2, I do expect a well sustained revision and comments of the points highlighted.

We believe that our revised manuscript addresses every issue raised by R2, who is fundamentally concerned with the resolution, depth of focus, and spatial sampling frequencies of the microscopes used in our study. In particular, R2 suggests that the camera used on the commercial microscope might not adequately sample the optical field, which may limit the potential improvements in classification performance one might expect from using shorter wavelengths exhibiting improved diffraction-limited resolution.

To investigate this and other related topics, we acquired Point Spread Functions (PSFs) in the same brightfield modality as all the experimental images in the study and included them as a supplementary figure. Curve fitting was used to extract basic parameters such as lateral and axial resolution in each imaging condition. These PSFs serve as a reference for several of R2's concerns, which we address in detail below. To this end, we have added additional content to the manuscript clarifying the technical factors limiting the performance of the commercial microscope in the near-UV regime.

I would also to have a clear discussion on the complexity of the problem in relation to the context of (in vitro specimens vs. clinical diagnosis specimens) as these pose different challenges.

The current method was not tested in a clinical setting, and we are clear on this point in the manuscript. However, the logical progression of these experiments would be the extension of

the method to clinical samples. We note that the leap to clinical samples will likely face several challenges. We refer the editor to lines 592-612 of the submitted manuscript for an enumeration of such anticipated complications, including imaging throughput, other cell types present in whole blood (lymphocytes, platelets, etc), the possibility of multiple malaria species, and RBC shape abnormalities. To further expand on these, we have added a key reference performing a broad, statistical overview ("The temporal dynamics and infectiousness of subpatent *Plasmodium falciparum* infections in relation to parasite density", Slater et al. 2019) and additional discussion of the sensitivities that are truly achieved in clinical practice, as quoted by this study.

I would consider to revisit the title from "High-performance automated classification of live P. falciparum can be achieved using ordinary brightfield microscopy: A superior alternative to labor-intensive fixation and staining." "Stain-free high-performance automated classification of live P. falciparum using brightfield microscopy" AND also consider be specific where you want to make the claim, eg clinical vs. biomedical use.

We are in agreement with the editor that the work is better represented by a simpler title with fewer implications. We have revised the full title as follows:

"Label-free imaging and classification of live *P. falciparum* enables high performance parasitemia quantification without fixation or staining."

And the short title as follows:
"Label-free imaging and classification of live *P. falciparum*"

We cannot make any decision about publication until we have seen the revised manuscript and your response to the reviewers' comments. Your revised manuscript is also likely to be sent to reviewers for further evaluation.

We look forward to receiving your evaluation after taking the improvements of the revised manuscript into consideration. We also thank the editor and reviewers for their helpful comments and feedback. We believe the resulting work is much higher in quality, and better-positioned in the context of the existing body of literature.

When you are ready to resubmit, please upload the following:

[1] A letter containing a detailed list of your responses to the review comments and a description of the changes you have made in the manuscript. Please note while forming your response, if your article is accepted, you may have the opportunity to make the peer review history publicly available. The record will include editor decision letters (with reviews) and your responses to reviewer comments. If eligible, we will contact you to opt in or out.

[2] Two versions of the revised manuscript: one with either highlights or tracked changes denoting where the text has been changed; the other a clean version (uploaded as the manuscript file).

Responses to reviewer 1:

The authors describe the development of an automated method and algorithms for identifying and counting red blood cells infected with P. falciparum. The stunning thing is that it can be done on life parasites. I cannot really comment much on the technical aspects of the method development and how much knowledge gain this actually represents, but the description is exceptionally detailed. I have no reason to believe that the method is not performing as described by the authors.

We thank reviewer 1 for recognizing the significance of performing classification on live cells, without the need for fixing and staining, and for their favorable opinion on the detail and rigor of our work.

What I can comment on however is the claim that this method would make standard malaria microscopy obsolete. I think this is quite grossly overstated.

This is an important point. Nowhere in the manuscript do we make the claim that this method, as described, renders manual malaria microscopy obsolete. Within the manuscript we are careful to point out the future challenges that must be overcome for clinical utility (lines 592-612 of the original manuscript).

That said, our original manuscript contains forward-looking statements, suggesting that at the current pace of technological improvement, label-free imaging and counting should one day replace the manual counting. We have modified all such statements (Abstract, Author Summary, and Conclusions) to not insinuate that the current method, as described, would replace current field methods. Further technological advances and clinical field utility studies would be required for a claim of superiority, and that remains to be done.

The updated conclusions paragraph reads as follows:

"The past decade has witnessed ongoing technological improvements in machine learning and low cost, high performance imaging and processing. With additional advances in these areas, implementation of label-free imaging and classification of live *P. falciparum* will enable major reductions in technician time, training, reagent cost, and observer variability with respect to the analysis of parasitized blood samples around the world, in both laboratories and ultimately in the field, although rigorous clinical utility studies will be required. Our results strongly suggest that advances in label-free imaging should enable rapid, automated, and highly accurate clinical diagnosis of fresh, whole blood – replacing error-prone and labor-intensive manual slide preparation and counting, a process that has changed little in over a century."

We have also made the following changes in order to rectify any remaining ambiguity:

1. The title has been simplified and implications removed.
2. We have added statements in the introduction and discussion highlighting that to match the absolute sensitivity of *thick* blood smears (whether in laboratory or in clinical settings) will require improvements in imaging throughput.

3. For the limited claims regarding thin blood smears, we have added emphasis that this is presently only tested in laboratory settings (not clinical).

Right now, as far as I understand, the automated method is able to achieve limit of detection of around 0.1% parasitemia (abstract). This is nowhere near the limit of detection achieved by standard light microscopy for malaria diagnosis, which uses thick blood films and has a limit of detection of around 10 (5-20) parasites per microliter of blood [1, 2]. Even though it is not entirely appropriate, for illustrative purposes, we can convert this to parasitemia based on the assumption that one microliter of blood has around 5 million red cells. Thus, the LOD of gold standard, expert light microscopy is on the order of around 0.0002% parasitemia and thus literally a thousand-fold better than the automated method described. So the new method would have to be able enumerate several million cells in around 30 minutes to be able to compete with standard light microscopy in terms of time to diagnosis and limit of detection.

The goal of the current work was to establish label-free imaging and parasite recognition limited to thin smears only. To support this limited claim regarding thin smears, we referenced a rigorous study comparing the accuracy of hundreds of trained microscopists scoring clinical thin blood smears that have each been characterized by expert consensus, and overlay the variation of microscopists in the study over a similar range of parasitemia (Figure 8 and ref 10, Manser et al). The referenced study exhibits similar variation as our own technicians' manual counting. We additionally referenced and compared with the WHO standard competence scale (lines 588-591 and ref 11), the highest level of which requires scoring within 25% accuracy only 50% of the time.

However, to put future extensions of our method into the context of thick blood smear sensitivity, we additionally added a reference and discussion of a broad, statistical overview (Slater et al. 2019) of clinical microscopy performance, including thick smears. This comprehensive review and analysis of clinical microscopy is an important benchmark. We have added additional text to compare our results with benchmarks and performance envelopes described here. We note that these authors calculated (from their meta-survey across many studies) that there is only a median probability of 29.7% that trained manual microscopy technicians will detect parasites at 100 / uL (0.002%), and only 60% probability of detection at 1000 / uL (0.02%). See figure 2I in Slater et al (2019).

So, while examples of true positive detection by microscopy as low as 0.0002% parasitemia certainly exist, what Slater et al. rightly point out is that the stated LODs do not take into account what is missed, and that the actual probability of correctly detecting parasites even at 1000 / uL (0.02%) is close to the flip of a coin. The specific text of our modification on this point is conservative and recognizes that the current method will need to advance further.

Our updated discussion statement addresses this:

on embedded systems at high speeds. However, such a system would likely encounter several challenges. First, clinical parasitemia levels vary widely [44,47], and must be distinguished from healthy with a high degree of confidence. As a result, clinical diagnostic procedures typically include both thick and thin blood smears. Thick blood smear sensitivity also varies widely between reports [44]. A comprehensive statistical analysis of the literature [44] quotes a median probability of 29.7% that trained manual microscopy technicians will detect parasite levels of 100/μL, and similarly, can detect 1000/μL with 60% probability – the latter being approximately equivalent to 0.02% parasitemia, or slightly below the lowest dilution point in our titration (0.036%). Future extensions of this method will therefore require increased imaging throughput to match the sensitivity of clinical thick blood smears. Second, individuals may be infected with more than one *Plasmodium* species (we did not perform speciation in this work),

Given the complexity of the instrumentation and computational resources (in particular in view of resource limited settings where malaria is important), it is hard to imagine this to rapidly transition to something used for malaria diagnosis in Africa.

Nevertheless, given the rapid gains made with AI and computational power, it may be possible that in a few years time the required numbers of cells can be enumerated. As such, the question whether to accept (with major revisions) or reject this manuscript is probably about whether the AI development and advance made in technology represent a knowledge gain that is significant enough to warrant publication, as this has no impact on clinical or laboratory practice in the near future.

I am not able to judge on this part but can highlight that there are other automated microscopy methods that have been proposed in the past so the idea is certainly not new and there are many papers out there (I just give one example here).[3]

In the case of the custom UV microscope, we wholeheartedly agree with the reviewer's opinion and do not anticipate wide adoption of microscopy using deep UV wavelengths. However, our demonstration of the method on a standard commercial brightfield microscope represents a dramatic reduction of complexity and barrier to usage as compared with other published methods (the introduction references many examples). In fact, our method can be adopted without any modification to a standard microscope, or with the simple addition of a 405 nm light source to access increases in performance.

Regarding the computational resources, we are fortunate enough to live in the midst of a revolution in low-cost, embedded, computation hardware specialized for deep model inferencing. There are many examples of sufficiently portable computer hardware devices under $100 (Google Coral, Jetson Nano, etc...). In our opinion, this will not be a limiting factor for the extension of the method to diagnostic settings, given the growing availability of low-cost computation hardware. However, several other anticipated challenges are indeed expected (lines 592-612).

The utility of our method lies in the elimination of the fixation and staining steps, saving ~45 minutes, costly reagents, associated variability in stain quality, and removal of the inconsistency

in counting proficiency across different technicians. Establishing consistent and repeatable performance statistics in parasitemia quantification will represent a substantial advance: despite the high sensitivity of thick blood smears, both Manser et al. (2013) and Slater et al. (2019) highlight the true variability that is present in manual microscopy; the former in relation to expert consensus, and the latter to PCR. The proof of concept reported here breaks new ground in eliminating the above sources of variability, is immediately applicable in biomedical laboratory applications, and can serve as a foundation for future advances into the clinical diagnostic field. Distinct from the array of other methods reported in this field, we present the first evidence that label-free parasitemia analysis can be achieved by the application of deep learning with a regular microscope.

But doing it with life cells from fresh blood is certainly a step forward and I feel that the presentation of the method that the authors put together in their manuscript is excellent.

We thank the reviewer for their positive reflection on the presentation of our method. We hope that the revisions presented here increase clarity for readers, particularly in the matter of the scope of validity of the method as described, how it relates to the existing literature, and the future applications it might enable (with their associated challenges overcome).

1. World Health Organisation: Basic malaria microscopy. 2nd ed edition. Geneva. (2010).

2. Schneider, P. et al. (2004) Quantification of Plasmodium falciparum gametocytes in differential stages of development by quantitative nucleic acid sequence-based amplification. Mol Biochem Parasitol 137 (1), 35-41.

3. Ross, N.E. et al. (2006) Automated image processing method for the diagnosis and classification of malaria on thin blood smears. Med Biol Eng Comput 44 (5), 427-36.

Reviewer #2: the review is given as an attached file

Have all data underlying the figures and results presented in the manuscript been provided?

Large-scale datasets should be made available via a public repository as described in the *PLOS Computational Biology* data availability policy, and numerical data that underlies graphs or summary statistics should be provided in spreadsheet form as supporting information.

Reviewer #1: Yes

Reviewer #2: Yes

PLOS authors have the option to publish the peer review history of their article (what does this mean?). If published, this will include your full peer review and any attached files.

If you choose “no”, your identity will remain anonymous but your review may still be made public.

Do you want your identity to be public for this peer review? For information about this choice, including consent withdrawal, please see our Privacy Policy.

Reviewer #1: No

Reviewer #2: No

Figure Files:

While revising your submission, please upload your figure files to the Preflight Analysis and Conversion Engine (PACE) digital diagnostic tool, https://pacev2.apexcovantage.com. PACE helps ensure that figures meet PLOS requirements. To use PACE, you must first register as a user. Then, login and navigate to the UPLOAD tab, where you will find detailed instructions on how to use the tool. If you encounter any issues or have any questions when using PACE, please email us at figures@plos.org.

Data Requirements:

Please note that, as a condition of publication, PLOS' data policy requires that you make available all data used to draw the conclusions outlined in your manuscript. Data must be deposited in an appropriate repository, included within the body of the manuscript, or uploaded as supporting information. This includes all numerical values that were used to generate graphs, histograms etc.. For an example in PLOS Biology see here: http://www.plosbiology.org/article/info%3Adoi%2F10.1371%2Fjournal.pbio.1001908#s5.

Reproducibility:

To enhance the reproducibility of your results, we recommend that you deposit your laboratory protocols in protocols.io, where a protocol can be assigned its own identifier (DOI) such that it can be cited independently in the future. Additionally, PLOS ONE offers an option to publish peer-reviewed clinical study protocols. Read more information on sharing protocols at https://plos.org/protocols?utm_medium=editorial-email&utm_source=authorletters&utm_campaign=protocols

Reviewer 2:
The authors report an approach for performing automated classification of P. falciparum malaria parasites in thin blood smears using an ordinary brightfield optical microscope for acquisition

and a deep learning classifier for processing. The novelty and interest of the paper lie in the use of label-free imaging which enables the reduction of technician time and work, training, reagent cost and sample appearance variability. I think that such an approach using only ordinary brightfield microscopy would be of great interest for tackling the issue of malaria parasite identification, classification and diagnosis in agreement with the points raised before.

Overall, the paper is well-written and very detailed. The underlying idea consists in using deep and near UV illumination for which the blood and internal structures absorb more might subsequently leading to a stronger contrast (and better resolution due to a decrease of the wavelength) in the acquired images without resorting to staining. This idea was tested on two optical systems, one being in-house built and the other a standard inverted commercial microscope. Their results suggest that image classification performance depends upon both image resolution and contrast.

We are grateful to the reviewer for their positive reflection on the utility and detail of our work.

My comments are given below and would need to be addressed, especially the ones about the optical parts, before considering publication.

1) Regarding the analysis of the data, they compared the performance of their machine classifiers against human operation classification and the results provided seem to support and validate the usefulness of their approach. However, they used 2 different methods, one including 2 steps, namely segmentation and classification, and the other called Faster R-CNN for which classification is directly done onto the raw images. After arguing about the pros and cons of each method they say in line 436 that Faster R-CNN performs better than the other method. This brings the question of why both methods were used? Especially why bother with the additional segmentation step?

We thank the reviewer for the question. Indeed, in our ongoing work we opt for a strategy similar to the single-step method outlined above. However, the present manuscript under consideration consists of an expository overview and development of the method. We felt that including information encompassing what worked best, as well as what did not work best, were both important.

We also noted in the manuscript that training the Faster R-CNN network (single-step method) presented substantial logistical challenges in generating human-annotated labels -- we refer the reviewer to lines 419-435 in the submitted manuscript for a detailed account. In short, these challenges were overcome by usage of the two-step method itself, in order to provide enough training examples for the one-step method. Hence, the two-step method was an integral part of the process, on which the one-step method depended. We therefore decided the results of this method were central to the paper.

2) At the beginning of the paper, the authors provide a state of the art about methods enabling identification and detection of malaria parasites with high resolution. Among them they refer to flow-cytometry, differential interference contrast, optical diffractive tomography, quantitative phase microscopy and digital holographic microscopy. The author forgot to mention Fourier Ptychographic Microscopy (FPM) which is a new low-cost computational imaging technique,

phase sensitive and providing large field of view and high resolution simultaneously [Ou et al., “High numerical aperture Fourier ptychography: principle, implementation and characterization,” Optics Express 23, (2015)]. It has also proved its ability for rendering 3D information (over different focal planes) into a single 2D image so that all the fine details of the sample were retained, with examples applied to thin blood smears and detection of malarial parasites [Rémy Claveau et al., "Digital refocusing and extended depth of field reconstruction in Fourier ptychographic microscopy," Bio. Opt. Express 11, (2020)]. Finally, this technique was also coupled to a classification network to image blood smears and provide an automatic malaria parasite detection and classification [Kim et al., “Multi-element microscope optimization by a learned sensing network with composite physical layers,” 45, (2020)]. I strongly advise the authors to have a look at this paper.

We thank the reviewer for informing us of additional past work of high relevance. We have updated our manuscript to include references to these papers.

3) As they use deep UV light, much more energetic than IR illumination usually used to image biological samples, I was wondering whether there would be a bad side effect on the cells?

Although continuous live cell imaging has been demonstrated with deep UV light (reference [33] in the submitted manuscript), it is generally accepted that these wavelengths are indeed highly phototoxic to living cells. In our own anecdotal experience, without special attention paid to minimize exposure, cells do not survive long under deep UV irradiation. However, we would suggest that in the context of parasitemia analysis, that phototoxicity to the cells is not a factor affecting the conclusions drawn in this paper. After all, the cells are bleached immediately after the imaging experiment.

4) For the segmentation of the RBCs they used a ResNet-50 network which was trained by manually segmenting RBCs with diverse appearances. As the cells display strong membrane contrast, was there a real benefit of using a neural network compared to a classic Otsu binarization method?

We thank the reviewer for the insightful question. Classical segmentation is indeed effective in identifying the large majority of red blood cells in our images. However, in the case of late-stage parasites, much of the cytoplasmic hemoglobin has been consumed and re-allocated to the condensed crystal form. As a result, cells with late-stage parasites are often not properly identified as RBCs by simple thresholding because the resulting binary region is small, and difficult to distinguish from acellular debris. Training a deep network for this purpose established a broader capability for detection of RBCs containing parasites of all stages and contrast levels.

Finally, my main concerns are related to the optical parts of the paper. I think there is a lack of important information, especially regarding the spatial resolution they achieve with both systems and the description of the cameras. There is also a serious confusion about the relationship between resolution, magnification, depth of field and numerical aperture of an objective lens, which might disprove some of their analysis.

The reviewer makes important points regarding the effect of wavelength and numerical aperture on resolution and depth of focus, and alludes to the Nyquist criteria, which determines the camera pixel size required to avoid aliasing and loss of high-frequency optical information. We address their specific concerns below.

4) Fig. S6: I do understand the cell contrast they get using UV light given the higher absorption but I'm quite surprised by the contrast with visible light illumination given that no staining is used. Maybe the spectrum of the source would help understand better. I assume it to have a wide spectrum compared to the other UV LED.

The reviewer highlights a very interesting phenomenon. Namely, that parasite contrast depends on both the real and imaginary portions of the refractive index, that these components vary across the various cellular compartments, and that contrast changes as a function of focus. We discuss these phenomena (with specific references) in lines 334-345. In the case of live cells the plasma membrane remains intact, and appears to exhibit a substantially different real component of its refractive index as compared with the RBC. Most researchers/clinicians are habituated to looking at fixed blood smears where the membrane is not intact, and the refractive index is normalized with the host RBC. Anecdotally (not shown in this paper), we previously tried imaging fixed cells with no staining and found that contrast was poor, except for the pigmented hemozoin crystals. It is worth noting that a paper we reference (ref 15, Gruring et al) captures excellent, high-contrast images of live parasites using a high-end Zeiss 100×/1.4 oil immersion lens operating in DIC modality.

To address the reviewer's specific question about the spectrum of the commercial lamp, it is a white LED (we added this to the "Commercial microscope" subsection of the Methods). We also note that in the case of the visible LED used on the custom UV microscope, the spectrum is freely available online from the manufacturer (Thorlabs M565L3).

5) Fig S11 (description of the pipeline) and again in the Materials and methods section (paragraph "Two-step method"), they refer to "digital refocus". It might be a misuse of the term because digital refocusing requires the knowledge of the optical field (amplitude and phase) which they don't using a conventional brightfield microscope.

We thank the reviewer for pointing out our accidental misuse of this term. We have corrected our terminology, and clarified in the manuscript that our procedure simply refers to selection of the optimal focal planes during post-processing of the data.

6) The diagram of their optical system reveals that they do not use a Kohler illuminator. Hence, they can have some artefacts appearing on the image and further descreasing the resolution. Is there a reason behind?

The reviewer's comment refers to potential for spatial inhomogeneity associated with imaging the illumination source LED onto or near the sample plane. In contrast, Kohler illumination is a common technique in microscopy used to homogenize the spatial distribution of light at the sample plane by projecting the structure of the light source onto the aperture (Fourier) plane of the condenser; thereby trading off spatial non-uniformity for incidence angle non-uniformity. The reviewer's concern is pertinent, as our design parts from the more conventional Kohler illumination strategy.

We considered the tradeoffs of Kohler illumination: spatial uniformity would be improved at the expense of cost, complexity, and efficiency of transmission from source to sample plane. In short, we found that the emission from the LED die surface was spatially uniform and could be focused very near to the sample plane without deleterious illumination effects (the coefficient of variation was 4.5%, some of which likely arises from vignetting in the objective itself). In fact, the angular incidence spectrum in our setup is quite possibly *more* uniform than a typical Kohler

illumination system, given that the angular emission profile of the LEDs is broad and smooth, and was not given up as a tradeoff (as the reviewer also points out below, the numerical aperture of illumination is an important determinant of resolution). Our strategy therefore exhibits a highly-uniform angular incidence spectrum at the sample plane. A functional test of these claims is demonstrated with nearly-ideal point spread functions (PSFs) experimentally measured on our UV system (see S13-S14 Figs in the revised manuscript), confirming the effectiveness of our illumination strategy.

7) Throughout the paper they talk about "*maximal resolution achieved with this wavelength*"… However, they never give values (either theoretical or experimental) of the resolution they have although this represents an important information.

We address the concern by presenting a wavelength-dependent measurement of the Point Spread Functions (PSFs) on both our microscopes, which also addresses later concerns regarding sampling by the camera pixels. The results are summarized in a new table added as supplementary information. Our custom UV microscope indeed had the highest resolution of all configurations (114 nm half-width), as approximated by Gaussian fit.

In the Materials and methods section (paragraph "Deep UV microscope") they say that an adjustable iris was used to control the NA of the illumination. This directly affects the resolution, up to a factor of 2. Consequently it would be good to point out how much closed (open) the iris was.

To address this question, we have added more information to the "Deep UV Microscope" subsection of the Methods, concerning the diameter of the adjustable iris, which was kept fully open at a diameter of 12 mm near the back pupil of an f=25 mm aspheric quartz lens. The numerical aperture of the illumination was therefore:

$$NA = n * sin(\theta) = 1 * \frac{6}{\sqrt{(25^2+6^2)}} = 0.23$$

8) Other important information concern the camera. The authors should provide the specifications of the different cameras they used, in particular the spectral sensitivity and **the size of the pixel**. The latter is essential as it would give readers information about the actual field of view they can capture. Even more, it would indicate whether the resolution is optically limited (diffraction) or set by the size of the pixel… Intuitively, I'd say that there are no issues for the 100x/0.85 objective but I do have serious doubts about the sampling when using the 40x/1.3 objective (aliasing).

We have included the additional information regarding the characteristics of both cameras used in the study, and compared them to the experimentally-determined optical resolution. The requested optical parameters have been summarized in S14 Fig of the revised submission. The reviewer is correct in suggesting that the 40x/1.3 configuration would be aliased in the case of diffraction-limited imaging. However, our experimental PSF measurement of this microscope suggests that the incorrect coverslip thickness of our quartz flow cells resulted in substantial spherical aberration, leading to a loss of lateral resolution and artificially elongated depth of focus. The resulting optical system's resolution is therefore limited by aberrations, as opposed to camera pixel size or diffraction limit. We have updated the manuscript to indicate that in practice this is the case, that it could explain the lack of performance increase observed when reducing wavelength on the commercial microscope, and that eliminating these issues may lead to increased performance at 365 nm. Despite the non-ideality, we observe that the method is nonetheless effective on the regular microscope.

9) The authors trained their classifier using images at different focal planes, mainly to produce the label by selecting the slice for which the classification confidence was the highest. Why did they not use a common approach to solve this issue called Extended Depth of Field (EDoF) algorithms? Or was there a specific reason for not using it?

The reviewer's suggestion is interesting, and could potentially prove to be an avenue for future improvement over our approach. However, it was not clear to us whether such algorithms would function as intended in the optical regime we describe in the text (lines 329-346, figure 4b, and figure S13 in the original submission), where contrast inversion can be observed through focus. It is possible that some algorithms might capitalize on this effect. We did not attempt this type of analysis partly for simplicity, but also because live parasites undergo Brownian motion and dynamic conformation changes, rendering it difficult to register a sequential capture of a focal stack to the degree where the end result is improved. Such an analysis method would likely require simultaneous capture of multiple focal planes at the same time -- a feature not available to us at this time.

10) To capture the slices at different focal planes they used 0.5μm increments. This choice must be set according to the depth of field of the objective. But since the DoF is not provided, it is not clear why they picked that value?

Given the measured depth of focus 724 nm (at 285 nm wavelength) and step size 500 nm, the reviewer is correct that the optical field was not fully sampled. It is therefore possible that under ideal conditions, the maximum confidence focal slice selection method may have proven slightly more effective. We thank the reviewer for locating an opportunity for further improvement.

11) The capture of a focal stack was only performed for the custom UV microscope. Indeed, in lines 697-698, they state "*Images from the commercial microscope did not require re-focusing*". As the objective in the commercial microscope has an NA of 1.3 and the one in the UV microscope has a lower NA of 0.85, it should the other way round. Indeed, the lower the NA the greater the depth of field.

We agree with the reviewer that the diffraction-limited depth of focus would be smaller for NA = 1.3 than for NA = 0.85. However, our experimental measurement of each system's PSF under the imaging conditions used in the paper suggest that the opposite is actually true for the aberrated PSF, whose lateral resolution has been degraded and depth of focus substantially elongated as compared to the theoretical. This information is now included in revised S13-S14 Figs detailing resolution, depth of focus, and sampling.

The reviewer's comment highlights the need for increased clarity regarding the underlying reason for requiring z-stacks on the UV system as compared with the commercial system. Since our analysis on the UV scope compares classification performance as a function of focus, a consistent metric was required to normalize and define the 'best focus' plane of the sample. In lines 113-118 in the manuscript, we describe chromatic aberrations present in the quartz objective, which caused a focal shift between wavelengths. Focal stacks were therefore required in order to register the best focus planes across wavelengths. In the case of the commercial microscope, we found that the *de facto* depth of focus was large enough not to gain additional information by employing z-stacks. Of course, neither the resolution, nor presumably the classification performance were optimal as a result of the aberrations, which we have now made clear in the manuscript as a potential limiting factor for short wavelengths on the commercial microscope data.

12) Lines 378-382: "*We opted to acquire data with lower magnification than the 100×magnification on the custom microscope, using a 40×/1.30 oil immersion objective in order to acquire datasets with a larger numbers of cells. At 40×, we could acquire approximately 500-1,000 cells per field of view without without significant overlapping of cells, at* ***the cost of reduced resolution***".

I totally disagree with this claim. Indeed, the field of view with the 40x increases **but** the resolution gets better as the NA of the objective increases, not its magnification… The magnification can limit the resolution in case of there is an aliasing phenomenon, which also depends on the physical size of the pixel (see comment 8).

The reviewer's statement that the theoretical diffraction-limited spatial resolution depends on the numerical aperture of the microscope objective (not magnification) is relevant and correct. We agree with their insight, and refer R2 to our new measurement of the experimental PSFs and the table summarizing the results. We note that our statement as written remains correct due to diffraction-limited performance observed on our UV scope (with the correct coverslip), and non-ideal performance on the commercial microscope (with the incorrect coverslip). We acknowledge in the updated manuscript that improved performance on the commercial microscope could be achieved by resolving this issue in conjunction with employing a smaller camera pixel size.

13) Lines 393-396: "*Interestingly, images acquired at 365 nm, while expected to exhibit the highest nominal resolution on the commercial microscope, did not perform better than visible light. This is potentially explained by optical aberrations in the microscope objective in the near-UV regime, given that this trend was not observed using our custom UV microscope*"

I agree that optical aberrations in the near UV spectral range might be an explanation. However I highly suspect that there is also an issue related to insufficient sampling. If images at 365nm did not perform better than those acquired with visible light, it is maybe because the resolution was set by the pixel size of the camera, consequently decreasing the wavelength would not bring further improvement…

We refer the reviewer to the above discussion in points (7,8,10,11, and 12) regarding experimental PSFs, which addresses this related concern. We acknowledge that both spherical aberrations and camera pixel sampling would need to be addressed in order to fully capitalize on the potential improvements brought about by near-UV light on the commercial system.

We thank reviewer 2 for providing thorough feedback via their technical expertise in microscopy. We acknowledge that our use of a coverslip with non-ideal thickness caused degradation of resolution in the case of 365 nm and 405 nm on the commercial microscope, and that further improvements are certainly possible.

Nonetheless, we demonstrate that the functional test of classification performance across a large range of parasitemia remained accurate. Indeed, the main message of the paper is that it is possible to do this on live cells without fixation or staining, and without complex instrumentation, via the application of deep learning to these ordinary microscopy images.
