# Supplementary material for: Label-free imaging and classification of live P. falciparum enables high performance parasitemia quantification without fixation or staining
Source: PLoS Comput Biol. 2021 Aug 9;17(8):e1009257. doi: 10.1371/journal.pcbi.1009257 (PMC8376094; doi:10.1371/journal.pcbi.1009257)
Supplement: Attachment — Submitted filename: review.docx [file pcbi.1009257.s023.docx]

The authors report an approach for performing automated classification of P. falciparum malaria parasites in thin blood smears using an ordinary brightfield optical microscope for acquisition and a deep learning classifier for processing. The novelty and interest of the paper lie in the use of label-free imaging which enables the reduction of technician time and work, training, reagent cost and sample appearance variability. I think that such an approach using only ordinary brightfield microscopy would be of great interest for tackling the issue of malaria parasite identification, classification and diagnosis in agreement with the points raised before.

Overall, the paper is well-written and very detailed. The underlying idea consists in using deep and near UV illumination for which the blood and internal structures absorb more might subsequently leading to a stronger contrast (and better resolution due to a decrease of the wavelength) in the acquired images without resorting to staining. This idea was tested on two optical systems, one being in-house built and the other a standard inverted commercial microscope. Their results suggest that image classification performance depends upon both image resolution and contrast.

My comments are given below and would need to be addressed, especially the ones about the optical parts, before considering publication.

1) Regarding the analysis of the data, they compared the performance of their machine classifiers against human operation classification and the results provided seem to support and validate the usefulness of their approach. However, they used 2 different methods, one including 2 steps, namely segmentation and classification, and the other called Faster R-CNN for which classification is directly done onto the raw images. After arguing about the pros and cons of each method they say in line 436 that Faster R-CNN performs better than the other method. This brings the question of why both methods were used? Especially why bother with the additional segmentation step?

2) At the beginning of the paper, the authors provide a state of the art about methods enabling identification and detection of malaria parasites with high resolution. Among them they refer to flow-cytometry, differential interference contrast, optical diffractive tomography, quantitative phase microscopy and digital holographic microscopy. The author forgot to mention Fourier Ptychographic Microscopy (FPM) which is a new low-cost computational imaging technique, **phase sensitive** and providing **large field of view** and **high resolution** simultaneously [Ou et al., “High numerical aperture Fourier ptychography: principle, implementation and characterization,” Optics Express 23, (2015)]. It has also proved its ability for rendering 3D information (over different focal planes) into a single 2D image so that all the fine details of the sample were retained, with examples applied to thin blood smears and detection of malarial parasites [Rémy Claveau et al., "Digital refocusing and extended depth of field reconstruction in Fourier ptychographic microscopy," Bio. Opt. Express 11, (2020)]. Finally, this technique was also coupled to a classification network to image blood smears and provide an automatic malaria parasite detection and classification [Kim et al., “Multi-element microscope optimization by a learned sensing network with composite physical layers,” 45, (2020)]. I strongly advise the authors to have a look at this paper.

3) As they use deep UV light, much more energetic than IR illumination usually used to image biological samples, I was wondering whether there would be a bad side effect on the cells?

4) For the segmentation of the RBCs they used a ResNet-50 network which was trained by manually segmenting RBCs with diverse appearances. As the cells display strong membrane contrast, was there a real benefit of using a neural network compared to a classic Otsu binarization method?

Finally, my main concerns are related to the optical parts of the paper. I think there is a lack of important information, especially regarding the spatial resolution they achieve with both systems and the description of the cameras. There is also a serious confusion about the relationship between resolution, magnification, depth of field and numerical aperture of an objective lens, which might disprove some of their analysis.

4) Fig. S6: I do understand the cell contrast they get using UV light given the higher absorption but I’m quite surprised by the contrast with visible light illumination given that no staining is used. Maybe the spectrum of the source would help understand better. I assume it to have a wide spectrum compared to the other UV LED.

5) Fig S11 (description of the pipeline) and again in the Materials and methods section (paragraph “Two-step method”), they refer to “digital refocus”. It might be a misuse of the term because digital refocusing requires the knowledge of the optical field (amplitude and phase) which they don’t using a conventional brightfield microscope.

6) The diagram of their optical system reveals that they do not use a Kohler illuminator. Hence, they can have some artefacts appearing on the image and further descreasing the resolution. Is there a reason behind?

7) Throughout the paper they talk about “*maximal resolution achieved with this wavelength*”… However, they never give values (either theoretical or experimental) of the resolution they have although this represents an important information.

In the Materials and methods section (paragraph “Deep UV microscope”) they say that an adjustable iris was used to control the NA of the illumination. This directly affects the resolution, up to a factor of 2. Consequently it would be good to point out how much closed (open) the iris was.

8) Other important information concern the camera. The authors should provide the specifications of the different cameras they used, in particular the spectral sensitivity and **the size of the pixel**. The latter is essential as it would give readers information about the actual field of view they can capture. Even more, it would indicate whether the resolution is optically limited (diffraction) or set by the size of the pixel… Intuitively, I’d say that there are no issues for the 100x/0.85 objective but I do have serious doubts about the sampling when using the 40x/1.3 objective (aliasing).

9) The authors trained their classifier using images at different focal planes, mainly to produce the label by selecting the slice for which the classification confidence was the highest. Why did they not use a common approach to solve this issue called Extended Depth of Field (EDoF) algorithms? Or was there a specific reason for not using it?

10) To capture the slices at different focal planes they used 0.5µm increments. This choice must be set according to the depth of field of the objective. But since the DoF is not provided, it is not clear why they picked that value?

11) The capture of a focal stack was only performed for the custom UV microscope. Indeed, in lines 697-698, they state “*Images from the commercial microscope did not require re-focusing*”. As the objective in the commercial microscope has an NA of 1.3 and the one in the UV microscope has a lower NA of 0.85, it should the other way round. Indeed, the lower the NA the greater the depth of field.

12) Lines 378-382: “*We opted to acquire data with lower magnification than the 100×magnification on the custom microscope, using a 40×/1.30 oil immersion objective in order to acquire datasets with a larger numbers of cells. At 40×, we could acquire approximately 500-1,000 cells per field of view without without significant overlapping of cells, at* ***the cost of reduced resolution***”.

**I totally disagree** with this claim. Indeed, the field of view with the 40x increases **but** the resolution gets better as the NA of the objective increases, not its magnification… The magnification can limit the resolution in case of there is an aliasing phenomenon, which also depends on the physical size of the pixel (see comment 8).

13) Lines 393-396: “*Interestingly, images acquired at 365 nm, while expected to exhibit the highest nominal resolution on the commercial microscope, did not perform better than visible light. This is potentially explained by optical aberrations in the microscope objective in the near-UV regime, given that this trend was not observed using our custom UV microscope*”

I agree that optical aberrations in the near UV spectral range might be an explanation. However I highly suspect that there is also an issue related to insufficient sampling. If images at 365nm did not perform better than those acquired with visible light, it is maybe because the resolution was set by the pixel size of the camera, consequently decreasing the wavelength would not bring further improvement…
